# Comprehensive Mechanistic Characterisation of the Antidiabetic Profile of *Sutherlandia frutescens* Using Target-Directed In Vitro Assays and Cellomics

**DOI:** 10.3390/life16081348

**Published:** 2026-08-17

**Authors:** Nadine Pringle, Trevor C. Koekemoer, Maryna van de Venter

**Affiliations:** Department of Biochemistry and Microbiology, Nelson Mandela University, Port Elizabeth 6031, South Africa

**Keywords:** *S. frutescens*, *Lessertia frutescens*, diabetes

## Abstract

Several studies have suggested potential mechanisms through which *Sutherlandia frutescens* exerts antidiabetic effects, yet much of its therapeutic potential remains unexplored. This study aimed to provide greater insights into the antidiabetic capabilities and limitations of *S. frutescens* through the use of a comprehensive in vitro screening platform incorporating target-directed assays with automated image cytometry and analysis, where relevant. The antidiabetic effects of a crude hot aqueous extract of *S. frutescens* were assessed across well-characterised targets representing prominent hallmarks of diabetes: postprandial hyperglycaemia, insulin sensitivity, β-cell dysfunction, chronic inflammation and adipose tissue dysfunction. The findings revealed *S. frutescens* as a multicomponent therapeutic, with its individual target activities being relatively subtle and a few appearing novel. Notably, however, *S. frutescens* inhibited multiple targets relevant to postprandial hyperglycaemia—including carbohydrate digestion (29.1% and 26.4% inhibition for sucrose and maltose, respectively, at 500 µg/mL), intestinal glucose uptake in Caco-2 cells (between 9.51% and 20.92% reduction from 100 to 500 µg/mL) and protein glycation (23.2% inhibition at 200 µg/mL)—activities not previously documented in vivo. Glucose consumption in C3A hepatocytes showed a dose-dependent increase in glucose consumption from 12.5 to 100 µg/mL. Glucose consumption was reduced in palmitic acid-induced insulin-resistant L6 skeletal muscle cells from 100% to 69.57%, while 50 µg/mL *S. frutescens* restored it to 97.84%. *S. frutescens* also enhanced INS-1 β-cell survival under oxidative stress at concentrations as low as 12.5 µg/mL. The integration of automated image cytometry and analysis provides a novel approach with which to characterise its antidiabetic properties and elucidate its potential molecular mechanisms. Overall, *S. frutescens* is shown to impact several interrelated mechanisms simultaneously, suggesting that the coordinated modulation of multiple pathways may contribute to its overall biological activity. Whether these effects are additive or synergistic remains to be determined.

## 1. Introduction

Diabetes mellitus refers to a group of chronic endocrine metabolic disorders primarily characterised by hyperglycaemia due to defects in insulin action, secretion or both. Diabetes affects carbohydrate, protein and lipid metabolism while simultaneously disrupting the body’s electrolyte balance and is thus associated with a host of both long- and short-term complications, which can affect almost every organ system in the body [1,2].

Currently, several treatment strategies aimed at regulating blood glucose levels in an attempt to mitigate the long-term complications associated with diabetes exist; however, severe adverse reactions, cost and accessibility remain impeding factors—particularly to those living in low- and middle-income countries. Consequently, traditional medicines have gained increasing attention as a plausible solution for improving diabetes management [3].

The characterisation of the antidiabetic efficacy of medicinal plants remains extremely challenging due to both the multifactorial nature of diabetes as well as the extreme complexity of plant extracts. As a result, many in vivo studies investigating the antidiabetic potential of medicinal plants appear to have selected animal models based on availability and/or popularity, as the precise therapeutic target(s) of plant extracts are rarely understood from existing traditional knowledge. Consequently, our knowledge and understanding of the antidiabetic properties of these plants remain highly fragmented and incomplete [4].

Commonly referred to as the cancer bush, *Lessertia frutescens* (L.) Goldblatt & J.C. Manning, previously named and still most frequently referred to as *Sutherlandia frutescens* (L.) R.Br., is classified as part of the Fabaceae family, which forms an attractive shrub that can grow up to 2 metres in height. It has a silvery appearance, which is due to the presence of hairy leaves, and large red flowers that give rise to paper-like seed pods. The leaves of *S. frutescens* contain several bioactive ingredients, including canavanine, γ-aminobutyric acid (GABA), pinitol, flavonoids, polysaccharides, amino acids and triterpenoids. The major cycloartane-type triterpenoid saponin commonly found in commercial materials is SU1 (=Sutherlandioside B) [5,6].

In South Africa, *S. frutescens* has been used as a traditional medicine by various healers to treat diabetes using aqueous extracts or infusions from leaves and stems [7,8,9]. The reported antidiabetic activities of *S. frutescens* include preventing insulin resistance in Chang liver cells by promoting glucose uptake and preventing steatosis [10], increased glucose utilisation in C2C12 myocytes by stimulating GLUT-4 translocation to the plasma membrane via the PI3K/Akt pathway, promoting INS-1 pancreatic β-cell proliferation and insulin secretion [11], and stimulating glucose consumption while simultaneously reducing triglyceride accumulation in 3T3-L1 adipocytes [12]. Furthermore, GABA was shown to promote human β-cell proliferation and exert anti-apoptotic effects [13]. GABA was also found to restore β-cell mass and effectively reverse the disease in mouse models of T1D [14]. *S. frutescens* has also previously demonstrated antioxidative and anti-inflammatory properties, amongst others [15,16,17,18].

Although limited, in vivo studies have explored the antidiabetic potential of *S. frutescens*. An aqueous shoot extract resulted in significant hypoglycaemia in a streptozotocin-induced rat model [19], while crushed leaves in the drinking water of pre-diabetic rats sustained on a high-fat diet (HFD) successfully normalised fasting insulin levels, enhanced peripheral tissue glucose uptake and suppressed intestinal glucose uptake [20]. A commercial extract of *S. frutescens* was also shown to successfully prevent the development of insulin resistance by reducing plasma free fatty acid levels in a HFD rat model [21]. Additionally, *S. frutescens* decreased plasma insulin, free fatty acid and triglyceride levels in a HFD-induced insulin-resistant rat model [12], and was subsequently suggested to have potential as a therapeutic strategy for T2D and obesity.

While these in vivo studies support the antidiabetic activity of *S. frutescens* in a biologically complex setting, they are severely limited as they do not address the full domain of potential antidiabetic properties. It is well recognised that no single animal model recapitulates all of the characteristic features of human diabetes; rather, each individual model is restricted to the specific molecular background employed to achieve a state representative of diabetes [22]. For example, models which are based on a HFD develop moderate insulin resistance and elevated glucose levels, but they typically do not develop basal hyperglycaemia and therefore relate poorly to overt diabetes. In contrast, models which destroy pancreatic β-cells (such as those using alloxan and streptozotocin) develop strong hyperglycaemia, but retain full insulin sensitivity. Furthermore, differences in experimental parameters, such as HFD formulations and duration of the experimental protocol, also impact the pathogenic outcome of a model. Early on during dietary interventions, some pathogenic markers plateau or even dissipate completely, for example hyperglycaemia, while others progressively worsen over the time course of the experimental protocol, such as insulin resistance and chronic inflammation [23].

Therefore, selecting an appropriate animal model and experimental design to demonstrate the potential efficacy of plant extracts requires prior knowledge of at least the most prominent antidiabetic mechanism(s), a requirement that is not met based on ethnobotanical information alone. Furthermore, medicinal plants are multicomponent therapeutics, raising the potential of engaging with multiple and diverse therapeutic mechanisms simultaneously, a situation poorly compatible with standard animal models of diabetes, as highlighted above. This raises concerns as to the capacity of popular in vivo models to truly define the clinical potential of antidiabetic medicinal plants and the realisation that meaningful antidiabetic properties could go unnoticed if such mechanisms are not incorporated in the animal model selected for experimentation. These limitations suggest that, for complex multicomponent therapeutics such as medicinal plant extracts, broad mechanistic characterisation should precede animal experimentation. Establishing the spectrum of biological activities in vitro provides a rational basis for selecting the most appropriate in vivo model and experimental design, thereby increasing the likelihood that therapeutically relevant mechanisms are adequately represented.

Recent advances in cellomics—a branch of cell culture that integrates high-throughput screening with quantitative cell analysis—have enhanced predictive capacity, enabling target-directed in vitro screening to fully characterise the antidiabetic capabilities and limitations of medicinal plants. This characterisation serves to guide the selection of the most appropriate animal model for subsequent studies. The accuracy of such a screening platform would be largely determined by its comprehensiveness and ability to incorporate multiple potential therapeutic targets. Once its accuracy and reliability have been confirmed, extensive screening could rapidly provide the necessary data that would allow for the assignment of antidiabetic medicinal plants to suitable animal models with optimal experimental designs [4].

In the present study, we demonstrate the application of an integrated target-directed in vitro screening platform designed to provide a comprehensive mechanistic characterisation of medicinal plants with potential antidiabetic activity. The use of image cytometry and advanced image analysis software improves the accuracy and versatility of cell-based in vitro assays [24,25,26]. As a representative case study, the South African medicinal plant *S. frutescens* is evaluated across the major hallmarks of diabetes—including postprandial hyperglycaemia, insulin resistance, β-cell dysfunction, chronic inflammation and adipose tissue dysfunction—using 20 complementary biochemical and cell-based assays integrated with high-content imaging and computational image analysis. By generating a broad mechanistic profile before progression to animal studies, this strategy provides a more rational framework for identifying therapeutic strengths and limitations and the most appropriate in vivo models for subsequent investigation.

## 2. Materials and Methods

### 2.1. Reagents and Chemicals

DMEM, MEM, RPMI-1640, FBS and HyPure WFI Quality water were purchased from HighClone Laboratories, Inc. (South Logan, UT, USA). PBS and penicillin–streptomycin were acquired from Lonza (BioWhittaker, Verviers, Belgium). Antibodies for COX-2, iNOS and NF-κB were obtained from Cell Signalling Technology, Inc. (Danvers, MA, USA). Horse serum and 1% non-essential amino acids were purchased from Biowest, Nuaillé, France. All remaining chemicals and reagents were obtained from Sigma-Aldrich (St. Louis, MO, USA) unless specified otherwise.

### 2.2. Extract Preparation and Quality Determination

Leaves from *Sutherlandia frutescens* (L.) R.BR. subsp. *microphylla* (syn. *Lessertia frutescens* (L.) Goldblatt and J.C. Manning) were collected from Murraysburg, Western Cape, South Africa (−32.008025, 23.611365). A voucher specimen (PEU 14 800) is located in the herbarium of Nelson Mandela University (Port Elizabeth, South Africa) and was previously identified by Professor E. Campbell from the Botany Department. *Sutherlandia frutescens* (L.) R.Br. is an accepted name according to The Plant List database (http://www.theplantlist.org; accessed 1 July 2021).

A hot aqueous extract of *S. frutescens* was prepared using the method adapted from [16]. Briefly, dried plant leaves were grinded into a fine powder using a mortar and pestle. The powdered leaves were mixed with boiling deionised distilled water in a 1:10 (*w*/*v*) ratio for 30 min. Following extraction, the aqueous extract was centrifuged at 500× *g* (3000 rpm) for a period of 5 min at room temperature using an Eppendorf 5804R centrifuge. Using vacuum filtration, the supernatant was filtered twice through Whatman™ filter paper (no. 1) and the pellet was discarded. The extract was frozen at −80 °C, after which it was freeze-dried using VirTis SP Scientific sentry 2.0 freeze dryer (Gardiner, NY, USA). The extract was stored in a desiccator at 4 °C and wrapped in foil to protect it from light. For solubilisation, the extract was dissolved at a concentration of 40 mg/mL in DMSO and sterile HyPure WFI Quality Water in a 1:1 ratio prior to use. All stocks were prepared fresh on a weekly basis and stored at 4 °C.

Prior to biological evaluation, the quality and identity of the plant extract were verified by the Department of Pharmaceutical Sciences, Tshwane University of Technology, using a previously published UPLC-MS quality control protocol [27]. SU1, an accepted chemical marker for *S. frutescens*, was included as an authentic reference standard to confirm the quality and authenticity of the extract [27]. The analysis was performed as a quality control procedure and was not intended as a comprehensive phytochemical characterisation or structural elucidation of the extract. The hot water aqueous extract was dissolved in deionised water at a concentration of 1 mg/mL and sonicated for 15 min followed by filtration through a 0.2 µm syringe filter.

A UPLC analysis was conducted as reported previously [27], with slight modifications. Briefly, a Waters Acquity Ultra Performance Liquid Chromatographic system equipped with a PDA detector (Waters, Milford, MA, USA) and an Acquity UPLC BEH C18 column (150 mm × 2.1 mm, i.d., 1.7 μm particle size, Waters) maintained at 40 °C was used. A gradient elution method was employed with the flow rate set to 0.35 mL/minute. The mobile phase comprised solvent A (0.1% formic acid in water) and solvent B (acetonitrile) with a total run time of 18 min. The initial gradient of 80:20 (solvent A:solvent B) was maintained for 0.5 min (0–0.5 min) before changing to 40:60 (0.5–13.5 min), 0:100 (13.5–16 min), holding for 1 min (16–17 min), and finally returning to the initial 80:20 ratio for another 1 min (17–18 min). A 2 µL injection volume was used for all samples, and all data was captured and processed using Masslynx 4.1 chromatography software.

Mass spectrometry was conducted in negative ion electrospray mode with N_2_ as the desolvation gas. The desolvation temperature was set to 300 °C at a flow rate of 400 L/h, with the source temperature being 100 °C. The capillary and cone voltages were set to 2500 and 45 V, respectively, with data collected between 100 and 1500 *m*/*z*. The LockSprayTM interface was used during acquisition to ensure mass accuracy and reproducibility.

### 2.3. Cell Culture

All cell lines were purchased from Cellonex, with exception of human hepatoma-derived C3A hepatocytes (American Type Culture Collection, Manassas, VA, USA) and rat insulinoma-derived INS-1 pancreatic β-cells (donation from Prof. Rutter). Cells were cultured in 10 cm treated polystyrene petri dishes in DMEM or RPMI-1640 supplemented with 10% FBS and 1% (*v*/*v*) penicillin/streptomycin. Cultures were incubated in a humidified incubator at 37 °C with 5% CO_2_ and checked daily for contamination using an Axiovert 40C inverted microscope (Carl Zeiss, Jena, Germany). Cells were sub-cultured when approximately 80% confluency was reached and culture medium was replaced every 3–4 days to encourage optimal growing conditions.

### 2.4. Basal Cytotoxicity Screening and Selection of Concentration Ranges

The extract concentrations used in this study were selected to reflect plausible differences between intestinal and systemic exposure following oral administration. Intestinal targets were evaluated at concentrations up to 500 μg/mL because phytochemicals may transiently attain relatively high local concentrations within the gastrointestinal lumen prior to absorption, metabolism, and elimination. For example, an oral dose of several hundred milligrams dispersed within approximately 1 L of gastrointestinal fluid could theoretically produce luminal concentrations in the range of hundreds of micrograms per millilitre. In contrast, assays targeting systemic mechanisms were conducted at lower concentrations (≤200 μg/mL) to account for dilution within the blood volume, tissue distribution, and metabolic clearance, all of which substantially reduce circulating concentrations relative to those present in the gut. These concentrations should therefore be regarded as physiologically informed in vitro exposure ranges intended to model the expected gradient between intestinal and systemic availability rather than to represent actual plasma concentrations.

Cytotoxicity screening was conducted using Hoechst 33342/propidium iodide (PI) dual staining of cells as previously described [4]. All cells except the Caco-2 cells were seeded overnight into TPP tissue culture-treated 96-well microtiter plates using the following cell densities in complete medium containing 10% FBS: MEM with 1% non-essential amino acids for C3A hepatocytes (6 × 10^3^ cells/well), low-glucose DMEM for RAW 264.7 macrophages (3 × 10^4^ cells/well), RPMI-1640 for INS-1 pancreatic β-cells (2 × 10^4^ cells/well) and high-glucose DMEM for L6 myoblasts (3 × 10^3^ cells/well). Once the cells adhered to the 96-well microtiter plate, the culture medium was gently aspirated and the cells were treated with *S. frutescens,* up to a maximum concentration of 200 μg/mL for 24 h (pancreatic β-cells) or 48 h (C3A hepatocytes and RAW 264.7 macrophages).

Prior to treatment, the L6 myoblasts were first differentiated into myotubes. After seeding, the myoblasts were left to reach 90% confluence before replacing the cell culture medium with high-glucose DMEM supplemented with 2% horse serum. The culture medium was gently aspirated and replaced every 2–3 days for a total period of 6 days. On the 7th day, the cells were exposed to *S. frutescens* for 24 h.

The Caco-2 cells (5 × 10^3^ cells/well) were cultured in low-glucose DMEM and were seeded into 48-well TPP tissue culture-treated plates. The cell culture medium was replaced every 2–3 days for a total period of 5 days to allow the cells to reach confluence. The culture medium was then replaced with *S. frutescens* up to a maximum concentration of 500 μg/mL for 3 h.

After treatment, the cell culture medium was gently aspirated and Hoechst 33342/PI staining was achieved by incubating the cells in 50 μL of Hoechst 33342 (5 μg/mL in PBS) for 10 min at room temperature protected from light. An equal volume of PI (100 μg/mL in PBS) was added directly before image acquisition. Fluorescent micrographs were captured as described in Section 2.8 and used to determine the percentage of live and dead cells.

### 2.5. Intestinal Targets to Combat Postprandial Hyperglycaemia

#### 2.5.1. Alpha-Amylase Inhibition

The potential of *S. frutescens* to inhibit alpha-amylase enzyme activity was assessed as previously described by [28] using a colorimetric assay for the quantification of starch–iodine complexes. Briefly, 15 μL of test sample was pre-incubated with 5 μL porcine pancreatin (1 mg/mL in 1X PBS buffer solution; prepared on the day of use and stored on ice) in a 96-well microtiter plate for 10 min at 37 °C. The addition of 20 μL starch solution (2 mg/mL in distilled water; boiled with continuous stirring for 15 min; once clear, the solution was cooled to room temperature with continuous stirring and the volume of evaporated water was replaced) initiated the reaction, which was allowed to proceed for 30 min at 37 °C. The addition of 10 μL HCl (1 M in distilled water) halted the reaction and was followed by 75 μL of iodine reagent (0.127 g iodine and 0.083 g potassium iodide in 100 mL distilled water) to stain any remaining starch. The absorbance was measured at 580 nm using a BioTek PowerWave XS spectrophotometer (Winooski, VT, USA). No enzyme and no substrate controls were included for each sample, with acarbose (500 μM stock solution prepared in PBS) included as a positive control. The percentage α-amylase inhibition was calculated as follows:

$$\%\;\alpha \;-\;amylase\;inhibition\;=\;\frac{\left(amylase\;activity\;of\;control-amylase\;activity\;of\;test\;sample\right)}{amylase\;activity\;of\;control}\times 100$$

where amylase activity = A580 nm without enzyme − A580 nm with enzyme.

#### 2.5.2. Alpha-Glucosidase Inhibition

The method previously published by [29] was modified slightly and utilised to measure α-glucosidase inhibition using commercial rat intestinal acetone powder as an enzyme source. The intestinal α-glucosidase solution was prepared fresh on the day of use by adding 100 mg of acetone powder to 3 mL of phosphate buffer (0.01 M; pH 7.0). The solution was sonicated 12 times in an ice bath in 30 s bursts to prevent denaturing of the enzymes, followed by centrifugation at 1811× *g* (3000 rpm) at 4 °C for 20 min. The supernatant-containing enzyme was collected and stored on ice until use.

To prepare the reaction, 5 μL of test sample was pre-incubated in a 96-well microtiter plate together with 10 μL of sucrose/maltose (500 mM) and 75 μL maleate buffer (0.1 M; pH 6.0) at 37 °C for 5 min. The reaction was initiated by the addition of 10 μL rat intestinal α-glucosidase and allowed to proceed for 60 min at 37 °C. The glucose released into the solution was quantified spectrophotometrically using a modified version of the glucose oxidase/peroxidase method previously published by [30]. The reaction mixture containing maltose (5 μL) or sucrose (10 μL) was transferred into a new 96-well microtiter plate and incubated in the presence of 200 μL glucose oxidase reagent (3 mM phenol, 0.4 mM 4-aminoantipyrine, 0.25 mM EDTA and 2.5 U/mL horseradish peroxidase in 0.5 M PBS buffer; pH 7.0; 1 mU/mL glucose oxidase from Aspergillus niger was added directly before use) for 15 min at room temperature. The absorbance was measured spectrophotometrically at a wavelength of 510 nm and the percentage α-glucosidase inhibition was calculated using the following equation:

$$\%\;\alpha \;-\;glucosidase\;inhibition\;=\;\frac{\left(A405\;nm\;of\;control-A405\;nm\;of\;test\;sample\right)}{A405\;nm\;of\;control}\times 100.$$

#### 2.5.3. Intestinal Glucose Uptake in Caco-2 Cells

Caco-2 cells were seeded into a 48-well plate at a density of 5 × 10^3^ cells/well and left for approximately 5 days until they reached confluence, replacing the medium every 2 to 3 days. The cells were then exposed to the specified treatments (75 μL prepared in RPMI-1640 diluted to 8 mM glucose with PBS containing 0.1% BSA) for 3 h and the glucose uptake was measured by transferring 5 μL of the culture medium into a 96-well plate. Glucose oxidase reagent (200 μL prepared as described previously in Section 2.5.2) was added and the reaction was incubated for 15 min at room temperature, after which the absorbance was measured at 510 nm. The glucose consumption was normalised for the total cell numbers (Hoechst 33,342 staining) and the results expressed as a percentage of the control. Myricetin (0.5 mM), a known inhibitor of the intestinal glucose transporter GLUT-2 [31], was included as a positive control.

#### 2.5.4. DPP-IV Inhibition

Using the chromogenic substrate Gly-Pro p-nitroanilide hydrochloride (Gly-Pro pNA) together with recombinant human DPP-IV, the potential DPP-IV inhibitory activity of *S. frutescens* was assessed using a kinetic assay as described previously [3], with diprotin A (17.5 μg/mL) included as a positive control. Briefly, 14 μL of test sample prepared in Tris-HCl buffer (50 mM; pH 8.0) was pre-incubated with 0.2 μL undiluted enzyme in a 384-well plate for 5 min at 37 °C. The reaction was initiated by the addition of 20 μL substrate (20 mM Gly-Pro pNA stock solution prepared in DMSO; diluted to 0.2 mM with Tris-HCl buffer; pH 8.0), and the absorbance was measured spectrophotometrically at 410 nm every 3 min for a total period of 30 min. For each sample, the absorbance was plotted against time, with the linear portion of the slope used to calculate the percentage DPP-IV inhibition as below:

$$\%\;DPP-IV\;inhibition\;=\;\frac{\left(Gradient\;of\;control-gradient\;of\;test\;sample\right)}{Gradient\;of\;control}\times 100.$$

#### 2.5.5. Glycation Inhibition

The method previously described by [32] was used to measure the glycation inhibitory potential of *S. frutescens*. Using a 1.5 mL microcentrifuge tube, 250 μL BSA (400 μg) was combined with 240 μL glucose (200 mM) and 10 μL of test sample. All the samples were prepared in phosphate buffer (50 mM; pH 7.4) and heated to 60 °C in a heating block for 30 h. The samples were left to cool to room temperature, after which 100 μL was transferred into a new microcentrifuge tube and placed on ice. Following the addition of 10 μL of cold, undiluted tricarboxylic acid, the samples were agitated with a vortex and centrifuged at 4 °C for 4 min at 15,000× *g*. After discarding the supernatant, the pellet was dissolved overnight in 400 μL alkaline PBS (137 mM NaCl, 8.1 mM Na_2_HPO_4_, 2.68 mM KCl and 1.49 mM KH_2_PO_4_; pH 10.0). Following agitation to dissolve any remaining pellets, the samples were transferred into a 96-well plate (90 μL/well) and the fluorescence was measured by means of a Fluoroskan Ascent FL fluorometer (ThermoLabsystems, Kuopio, Finland) using the AGE excitation/emission wavelengths of 370/440 nm.

### 2.6. Targets for Insulin Resistance and Inflammation

#### 2.6.1. Hepatocyte Glucose Utilisation

The hepatocyte glucose utilisation was measured as previously described [4]. Using tissue culture-treated 96-well plates, C3A hepatocytes were seeded at a density of 1 × 10^4^ cells/well and left to attach overnight in complete cell culture medium consisting of MEM, 10% FBS and 1% non-essential amino acids (NEAAs). Following pre-incubation at 37 °C for 48 h in the presence of *S. frutescens* (0–100 µg/mL) or metformin (0.3 mM as positive control), the culture medium was gently aspirated and the cells were washed with 100 μL PBS containing calcium and magnesium (+Ca^2+^/Mg^2+^) to discourage cellular detachment from the 96-well plate. To measure glucose uptake, the addition of 25 μL incubation buffer (RPMI-1640 diluted in PBS containing 0.1% BSA to achieve a final glucose concentration of 8 mM) was followed by a further incubation period of 4 h at 37 °C.

The glucose remaining in the cell culture medium after 4 h was measured using the standard glucose oxidase/peroxidase method published by [30], and described in Section 2.5.2, using 5 µL of cell culture medium. The glucose consumption was calculated as a percentage of the control as follows:

$$Glucose\;consumption\;=\;\frac{\left(A510\;nm\;of\;cell-free\;wells-A510\;nm\;of\;test\;sample\right)}{\left(A510\;nm\;of\;cell-free\;wells-A510\;nm\;of\;control\right)}\times 100$$

where the control represents the cells without treatment, while the cell-free wells containing incubation buffer provides an indication of the initial glucose concentration.

#### 2.6.2. Inhibition of Hepatocyte Lipid Accumulation

Using palmitic acid to induce lipid accumulation in the C3A hepatocytes, the ability of *S. frutescens* to inhibit hepatocyte lipid accumulation was assessed using the method previously described by [4]. Using MEM supplemented with 10% FBS and 1% NEAAs, the C3A hepatocytes were seeded into tissue culture-treated 96-well plates at a density of 6 × 10^3^ cells/well at 37 °C and left to attach overnight. The culture medium was gently aspirated, and the cells were treated with *S. frutescens* (0–100 µg/mL) or metformin (3 mM as a positive control). All treatments were prepared in MEM supplemented with 2% BSA (fatty acid free) and 1% NEAAs (sterile filtered using a 0.1 μm syringe filter). To induce steatosis, 1 mM palmitic acid (stock solution prepared in absolute ethanol and diluted in culture medium; prepared fresh directly before use) was included in all treatments and the cells were incubated at 37 °C for 24 h.

Lipid staining was achieved using LipidTox Red for neutral lipids (ThermoFisher Scientific, Waltham, MA, USA) according to the instructions provided by the manufacturer. Briefly, formaldehyde was used to fix the cells by adding a final concentration of 4% directly to the culture medium. The cells were incubated for 15 min at room temperature, after which the medium was gently aspirated and the cells were washed with PBS (+Ca^2+^/Mg^2+^). The cells were incubated in staining solution (50 μL) containing Hoechst 33342 (5 μg/mL prepared in PBS) to enable identification of individual cells, as well as LipidTox Red (diluted 1:1000 to achieve a 1X solution), for 30 min at room temperature. The plates were wrapped in foil to prevent quenching of the fluorescent dyes. Fluorescent micrographs were captured as described in Section 2.8 and used to determine the mean stain area of LipidTox Red.

#### 2.6.3. Skeletal Muscle Glucose Utilisation and Inhibition of Lipid Accumulation

Using the protocol described in Section 2.4, L6 skeletal myoblasts were seeded into 96-well microtiter plates and differentiated into myotubes. The cells were then exposed to *S. frutescens* (0–100 µg/mL) or metformin (1 mM or 5 mM as positive control for glucose utilisation and inhibition of lipid accumulation, respectively) for 24 h at 37 °C. All treatments were prepared in high-glucose DMEM supplemented with 2% BSA (fatty acid free), and insulin resistance was induced by including 1 mM palmitic acid to the culture medium (prepared as described in Section 2.6.2). Glucose utilisation was measured as described for C3A hepatocytes (Section 2.6.1), with the incubation period reduced to 3 h. Lipid staining was performed using LipidTox Red as described for C3A hepatocytes (Section 2.6.2).

#### 2.6.4. Pancreatic Lipase Inhibition

Using the method previously described by [4], the potential porcine pancreatic lipase inhibitory activity for *S. frutescens* was assessed using the chromogenic substrate p-nitrophenyl palmitate (pNPP). To 10 μL of sample, 20 μL of porcine pancreatin (100 mg/mL prepared in 100 mM Tris-HCl; pH 8.0; the solution was centrifuged at 5000× *g* for 5 min with the supernatant used as the source of the enzyme; the solution was prepared fresh on the day of use and kept on ice) was added and the reaction was pre-incubated at 37 °C for 15 min. The reaction was initiated by the addition of 170 μL substrate solution, which was prepared by combining solution A, containing pNPP (1 mg/mL dissolved in isopropanol), with solution B, prepared in 100 mM Tris-HCl (pH 8.0) containing gum arabic (1 mg/mL), sodium deoxycholate (2 mg/mL) and Triton X-100 (5 μL per mL). The reaction was allowed to proceed for 25 min at 37 °C, after which the absorbance was measured spectrophotometrically at 405 nm. The percentage pancreatic lipase inhibition was calculated using the following equation:

$$\%\;lipase\;inhibition\;=\;\frac{A405\;nm\;of\;blank-A405\;nm\;of\;test\;sample}{A405\;nm\;of\;blank}\times 100.$$

#### 2.6.5. Immunofluorescent Detection of iNOS, COX-2 and NF-κB

The immunofluorescent detection of COX-2, iNOS and phosphorylated NF-κB were measured as described previously [4]. RAW 264.7 macrophage-like cells were seeded into sterile tissue culture-treated 96-well microtiter plates in low-glucose DMEM supplemented with 10% FBS at a density of 3 × 10^4^ cells/well and left to attach overnight at 37 °C. The culture medium was gently aspirated, and the cells were treated with *S. frutescens* (0–100 µg/mL) or resveratrol (50 µM as a positive control) as well as LPS (200 ng/mL) for the COX-2 and iNOS assays, and incubated for 24 h at 37 °C. For NF-κB, the cells were treated with *S. frutescens* (0–100 µg/mL) for 23 h at 37 °C followed by a further 1 h incubation in the presence of LPS (200 ng/mL).

Prior to staining, the cells were fixed using either formaldehyde (COX-2 and NF-κB) or the IntraPrep Permeabilization Reagent kit (Beckman Coulter, Brea, CA, USA). For iNOS, the cells were fixed and permeabilised using the IntraPrep Permeabilization Reagent kit according to the manufacturer’s instructions. For formaldehyde fixation, the cells were fixed in a fume hood by adding formaldehyde at a final concentration of 4% directly to the spent culture medium for 15 min at room temperature. The fixative was gently aspirated, and the cells were washed twice with 100 µL PBS (+Ca^2+^/Mg^2+^), after which the RAW 264.7 cells were permeabilised using 100 µL of ice-cold methanol and incubated for 10 min at −20 °C. Following gentle aspiration of the methanol, the cells were allowed to equilibrate to room temperature for 5 min in PBS.

Once the cells were fixed and permeabilised, they were washed twice with BSA (1% solution prepared fresh in PBS on day of use) followed by a 45 min incubation at room temperature in blocking solution (3% BSA solution containing 0.2% Triton-X 100 prepared fresh in PBS on day of use). Phospho-NF-κB p65 (Ser536)(93H1) Rabbit mAb, Cox2 (D5H5) XP Rabbit mAb (Alexa Fluor 488 Conjugate) and iNOS (D6B6S) Rabbit mAb (PE Conjugate) antibodies were prepared in 1% BSA/PBS using 1:800 dilutions. Following the gentle aspiration of the blocking solution, the cells were incubated in 50 μL of the specified antibody for 1 h at 37 °C. All antibody solutions and plates were wrapped in foil to prevent quenching of the fluorescent antibodies.

As the NF-κB antibody was unconjugated, staining with a secondary fluorescent antibody was required. The primary antibody was gently aspirated, after which the cells were washed twice with 100 μL of a 1% BSA/PBS solution and incubated in 50 μL of Anti-Mouse IgG (H+L) F(ab’)2 Fragment (Alexa Fluor 488 Conjugate) using a 1:1000 dilution for 60 min at 37 °C, and the reaction was protected from light. Fluorescent micrographs were captured as described in Section 2.8, with the exception that a 40X objective was used for NF-κB and a 20× objective for iNOS and COX-2.

### 2.7. Pancreatic Beta-Cell Function

#### 2.7.1. Calcium-Dependent Insulin Secretion

Using the method previously described by [4], INS-1 pancreatic β-cells were seeded into tissue culture-treated 96-well microtiter plates at a density of 1 × 10^4^ cells/well in RPMI-1640 medium with 10% FBS. The cells were incubated for 48 h at 37 °C, after which the medium was gently aspirated and the cells were loaded with the fluorescent dye Fluo-4 AM (ThermoFisher Scientific). The staining solution was prepared in PBS (+Ca^2+^/Mg^2+^) and contained Fluo-4 AM (2 μM) and Hoechst 33342 (5 μg/mL) to enable the identification of individual cells. The cells were incubated in 50 μL of staining solution at 37 °C for 45 min with all plates wrapped in foil to prevent quenching of the fluorescent dyes. This was followed by the gentle aspiration of the staining solution and a further 30 min incubation period in 50 μL PBS (+Ca^2+^/Mg^2+^) at 37 °C. An *S frutescens* hot aqueous extract (0–100 µg/mL) and tolbutamide (400 µM as a positive control) were prepared in PBS (+Ca^2+^/Mg^2+^) and added to the cells for 10 min at 37 °C. The fluorescent micrographs were captured as described in Section 2.8.

#### 2.7.2. Ferric Reducing Antioxidant Power (FRAP) Assay

The method previously described by [33] was used to conduct the FRAP assay. The FRAP reagent was prepared fresh on the day of use by mixing 20 mL sodium acetate buffer (300 mM), 2 mL TPTZ (10 mM), 2 mL ferric chloride (20 mM in distilled water) and 2.4 mL distilled water. In a 96-well microtiter plate, 50 μL of sample containing *S. frutescens* (0–200 µg/mL) or catechin (200 µM as positive control) was incubated with 200 μL of FRAP reagent for 30 min at 37 °C, after which the absorbance was measured spectrophotometrically at 593 nm. To obtain the gradient of the slope, the absorbance values were plotted against the concentration of *S. frutescens*. The results were then expressed as Trolox equivalents (TE) using the following equation:

*Trolox equivalents* (g/g) = *Gradient of trolox* ÷ *gradient of sample*.

#### 2.7.3. DPPH Free Radical Scavenging Assay

The DPPH radical scavenging ability of *S. frutescens* was measured using a previously described method [4]. Briefly, in a 96-well microtiter plate, 5 µL of sample was added to 120 µL of Tris-HCl buffer (50 mM; pH 7.4) and 120 μL of DPPH (0.1 mM prepared in absolute ethanol). The reaction was allowed to proceed for 20 min at room temperature, protected from light, after which the absorbance was measured spectrophotometrically at 513 nm. To calculate the percentage radical scavenging activity, the following formula was used:

$$\%\;DPPH\;scavenging\;=\;\frac{A513\;nm\;of\;control-A513\;nm\;of\;test\;sample}{A513\;nm\;of\;control}\times 100.$$

#### 2.7.4. TBHP-Induced Oxidative Stress

Using CellROX Orange reagent (ThermoFisher Scientific), the ability of *S. frutescens* to protect INS-1 pancreatic β-cells against oxidative stress induced by tert-Butyl hydroperoxide (TBHP) was assessed. Cells were seeded into tissue culture-treated 96-well plates at a density of 2 × 10^4^ cells/well and incubated overnight at 37 °C to enable cellular attachment. Following pre-treatment with *S. frutescens* or the positive control catechin (50 µM) for 24 h at 37 °C, TBHP (30 μM) was added directly to the culture medium containing the relevant treatments for 2 h at 37 °C to induce oxidative stress. After gentle aspiration of the culture medium, the cells were stained with PBS (+Ca^2+^/Mg^2+^) containing CellROX Orange reagent (5 μM) and Hoechst 33342 (5 μg/mL) for 30 min at 37 °C to enable the quantification of ROS and the identification of individual cells, respectively. The plates were wrapped in foil to prevent quenching of the fluorescent dyes. Fluorescent micrographs were captured as described in Section 2.8, with antioxidant activity determined using the mean stain area for CellROX Orange. The antioxidant activity measured using this model reflects the direct ROS scavenging effects (intracellular and extracellular) concurrently with potential changes in the inherent capacity of the cells to resist oxidative stress.

#### 2.7.5. Beta-Cell Proliferation and Promotion of Cell Survival

Pancreatic β-cell proliferation was measured using INS-1 cells. The cells were seeded in RPMI-1640 medium with 10% FBS at a density of 2 × 10^4^ cells/well into TPP tissue culture-treated 96-well plates and left to attach overnight at 37 °C. The culture medium was gently aspirated, and the cells were exposed to the relevant treatment conditions for 24 h. The total cell numbers were determined using Hoechst 33342 (5 μg/mL) staining as described in Section 2.4. Promotion of β-cell survival was assessed using the same protocol with the addition of TBHP (3.5 μM) in the cell culture medium to stimulate oxidative stress-induced cell death.

### 2.8. Imaging, Data and Statistical Analysis

All fluorescent micrographs were captured as described previously [4]. Using Molecular Devices ImageXpress Micro XLS Widefield Microscope for high-content analysis, fluorescent micrographs were obtained using the DAPI (Hoechst 33342), Texas Red (PI, CellROX Orange and LipidTox Red), FITC (Fluo-4 AM, NF-κB and COX-2) or TRITC (iNOS) filter sets. Nine image sites were acquired per well using a 10× magnification unless stated otherwise. MetaXpress 6.1 High-Content Image Acquisition and Analysis Software was used to analyse all micrographs using the Multi-Wavelength Cell Scoring application module. All experiments were conducted three times, each in triplicate, to enable a statistical analysis. Error bars were used to represent the standard deviation, which was calculated from the mean of three independent experiments. One-way ANOVA and Tukey HSD post hoc tests were used to compare the means of the data sets. Statistical significance was accepted at *p* ≤ 0.05 [34]. The Statistics Kingdom online calculator (ANOVA Calculator—One-way ANOVA and Tukey HSD test) was used to perform the statistical analysis.

## 3. Results

### 3.1. Phytochemical Analysis

**Figure 1:**
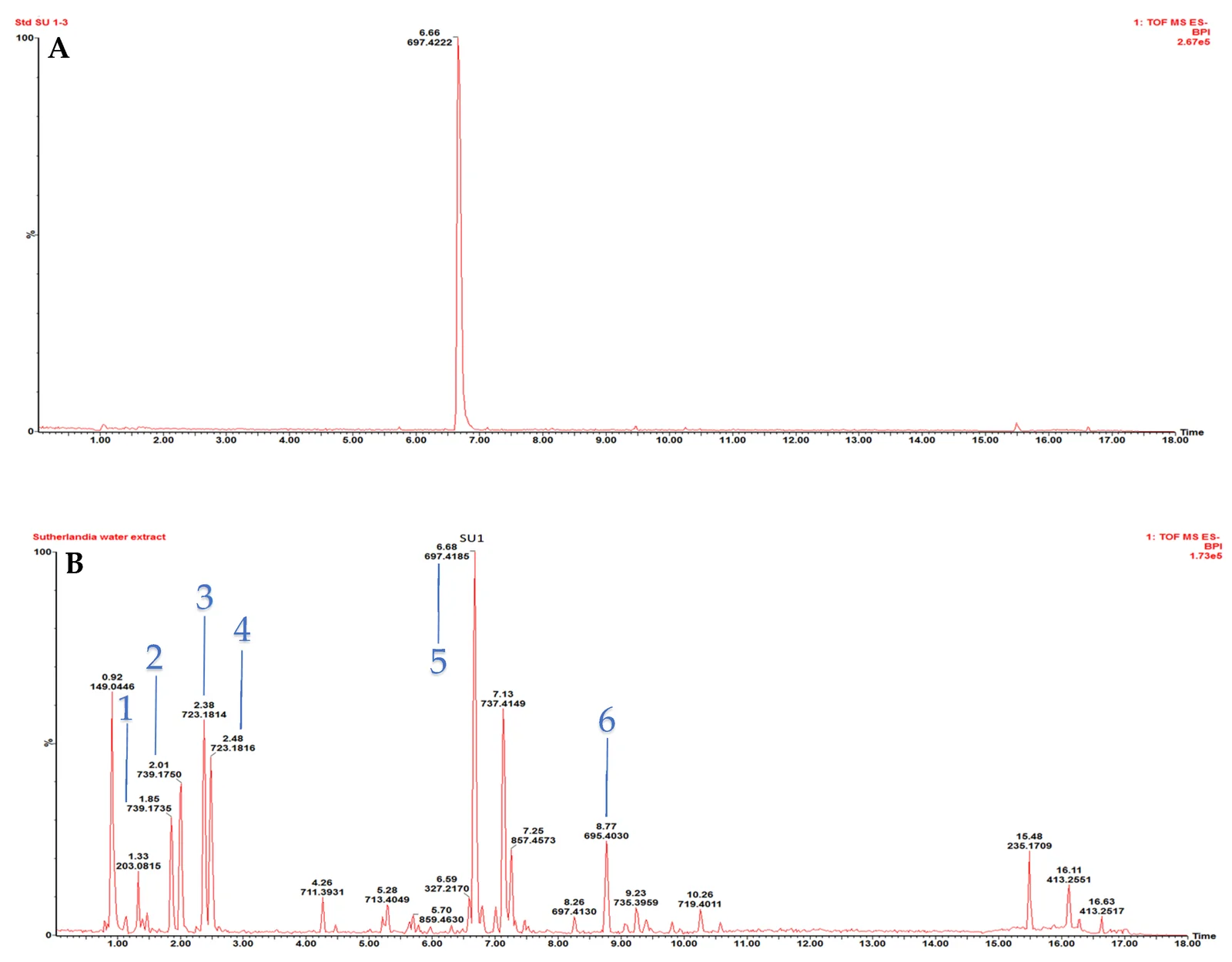
UPLC-MS chromatograms comparing (**A**) SU1 standard with (**B**) hot aqueous extract of *S. frutescens* from plant material used during the present study. The presence of four known flavanol glycosides (1 = Sutherlandin A; 2 = Sutherlandin B; 3 = Sutherlandin C; 4 = Sutherlandin D) and two known cycloartane glycosides (5 = Sutherlandioside B (SU1); 6 = Sutherlandioside C) can be confirmed. The chromatograms are presented to demonstrate the quality control fingerprint of the extract and confirmation of the SU1 marker compound; they are not intended as a comprehensive phytochemical characterisation of the extract.

The plant material used during the present study was confirmed to be of good quality during UPLC-MS, as the hot aqueous extract revealed SU1, with a retention time of 6.68 min (Figure 1B), to be a major peak. The chromatographic profile obtained was consistent with the characteristic fingerprint previously reported for *S. frutescens* [27,35]. In particular, the presence of SU1, confirmed using an authentic reference standard (Figure 1A), verified the quality and authenticity of the extract. Additional peaks were tentatively assigned to characteristic flavonol glycosides and cycloartane glycosides according to the established UPLC-MS protocol [27,35], and are presented to illustrate the characteristic phytochemical profile of the extract.

### 3.2. Basal Cytotoxicity Screening

The choice to use a hot aqueous extract of *S. frutescens* stems from the fact that this is the way that it would typically be prepared for use as a home remedy or for traditional use. Both the treatment concentrations and exposure or treatment time differed between cell lines. The extract concentrations were selected based on physiological relevance to the therapeutic target in question, with the maximum physiologically relevant extract concentration identified based on the guidelines suggested by [36]. As explained in Section 2.4, therapeutic targets which require drug absorption into the blood stream were tested at lower concentrations (200 µg/mL) to take into consideration the potential limitations of absorption, metabolism and greater dilution in the blood. Intestinal targets were tested at higher concentrations (500 µg/mL) due to the relatively smaller gut volume. The exposure or treatment time was dependent on the growth rate of the cell line, as experiments were ended when the cells reached approximately 80% confluence, to ensure that the effects of contact inhibition did not influence the results and to ensure sufficient quantities of cells for accurate image cytometry.

**Figure 2:**
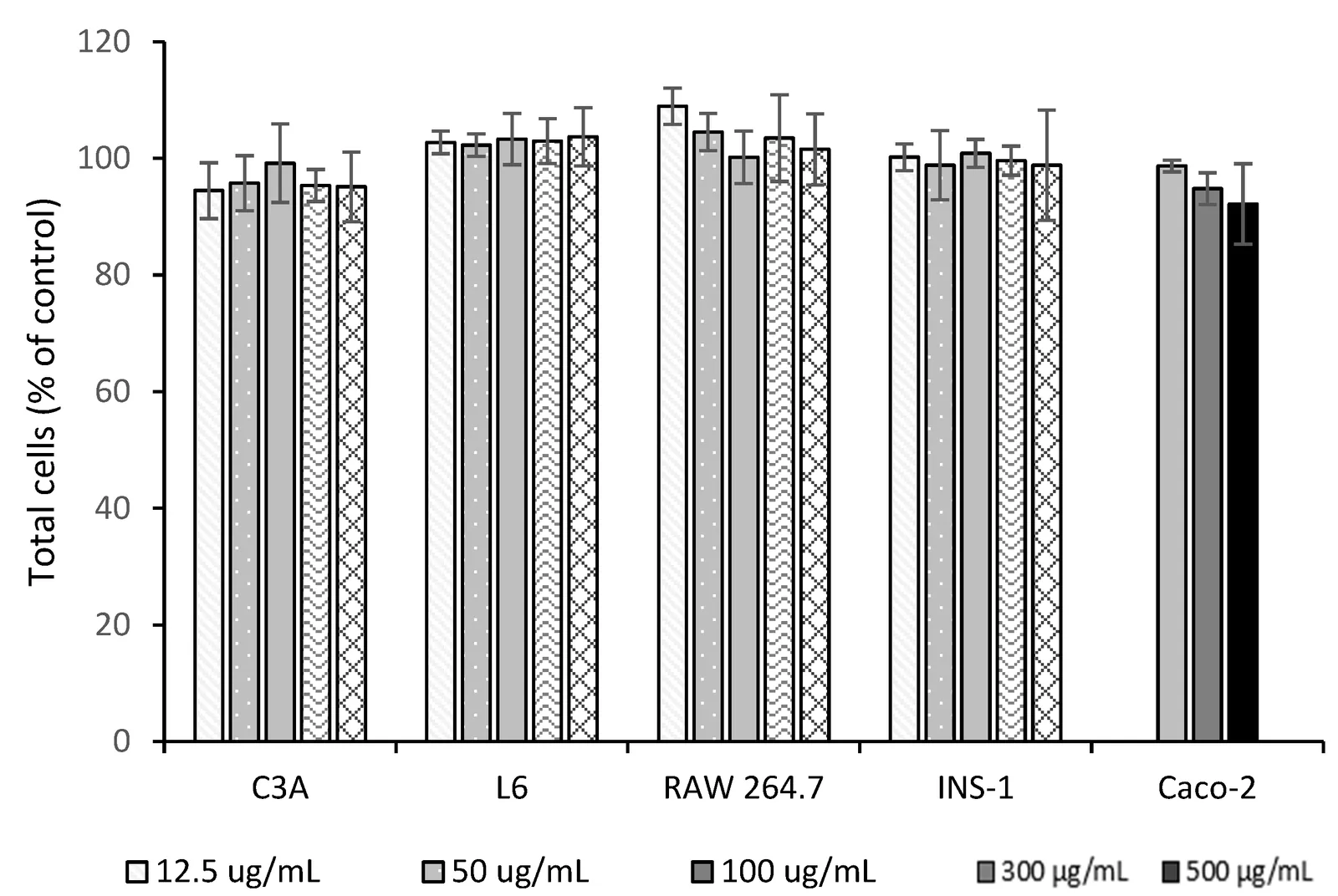
Basal cytotoxicity screening of *S. frutescens* hot aqueous extract comparing total cell numbers obtained using Hoechst 33342 staining of the nuclei for C3A hepatocytes after 48 h, differentiated L6 myotubes after 24 h, RAW 264.7 macrophages in the presence of LPS after 48 h, INS-1 pancreatic β-cells after 24 h and Caco-2 cells after 3 h. The results are reported as the mean ± standard deviation (error bars shown in black) with all experiments performed three times, each in triplicate (ANOVA showed no significant differences compared to the control).

Upon the evaluation of total cell numbers (Hoechst 33342 staining, Figure 2) and the population of dead cells (PI staining; Supplementary Material Figure S1) found for *S. frutecens* across five different cell lines, no significant decrease in the total cell numbers nor a significant increase in the percentage of cells that stained positive for PI were recorded. Consequently, *S. frutescens* did not display any significant toxicity in C3A hepatocytes, L6 skeletal myotubes, activated RAW 264.7 macrophage-like cells, INS-1 pancreatic β-cells or Caco-2 cells at the concentrations and exposure times tested. This indicates that the concentration range selected (0–500 µg/mL for Caco-2 and 0–200 μg/mL for all other cell lines) was well tolerated and unlikely to have influenced the accuracy of the selected antidiabetic screening assays.

The findings obtained here are congruent with a long history of traditional use and demonstrate why *S. frutescens* is generally considered to be safe [6,37]. Furthermore, a small randomised, double-blind clinical trial conducted in 2007 revealed that the consumption of 400 mg of the leaf powder of *S. frutescens* twice daily over a period of three months appeared to be well tolerated by healthy adults (clinicaltrials.gov, NCT00376415).

These findings appear distinctly different from the many in vitro reports claiming the anti-cancer activity of *S. frutescens* based on the cytotoxicity data generated from various cancer cell lines [38,39,40,41]. The concentrations required to reveal significant cancer cell toxicity in these studies ranged from 1.3 to 10 mg/mL, which far exceeds the concentrations used in the cell-based assays of the present study, as well as those recommended by [36]. Despite the myriad of in vitro studies claiming favourable cancer-specific mechanisms of toxicity, no in vivo studies corroborating any of these findings exist to date.

### 3.3. Intestinal Targets to Combat Postprandial Hyperglycaemia

One of the major challenges in diabetes management today remains the restrictions in controlling postprandial hyperglycaemia both effectively and consistently. Because starch amylolysis is accompanied by a substantial rise in plasma glucose levels, oral antihyperglycemic agents, such as α-amylase and α-glucosidase inhibitors, are frequently used during the early stages of T2D and in borderline patients in an attempt to prevent postprandial spikes in blood glucose levels [42,43]. This is achieved by inhibiting the action of carbohydrate-hydrolysing enzymes within the digestive tract, consequently reducing the rate at which glucose is absorbed into the blood stream [44].

**Figure 3:**
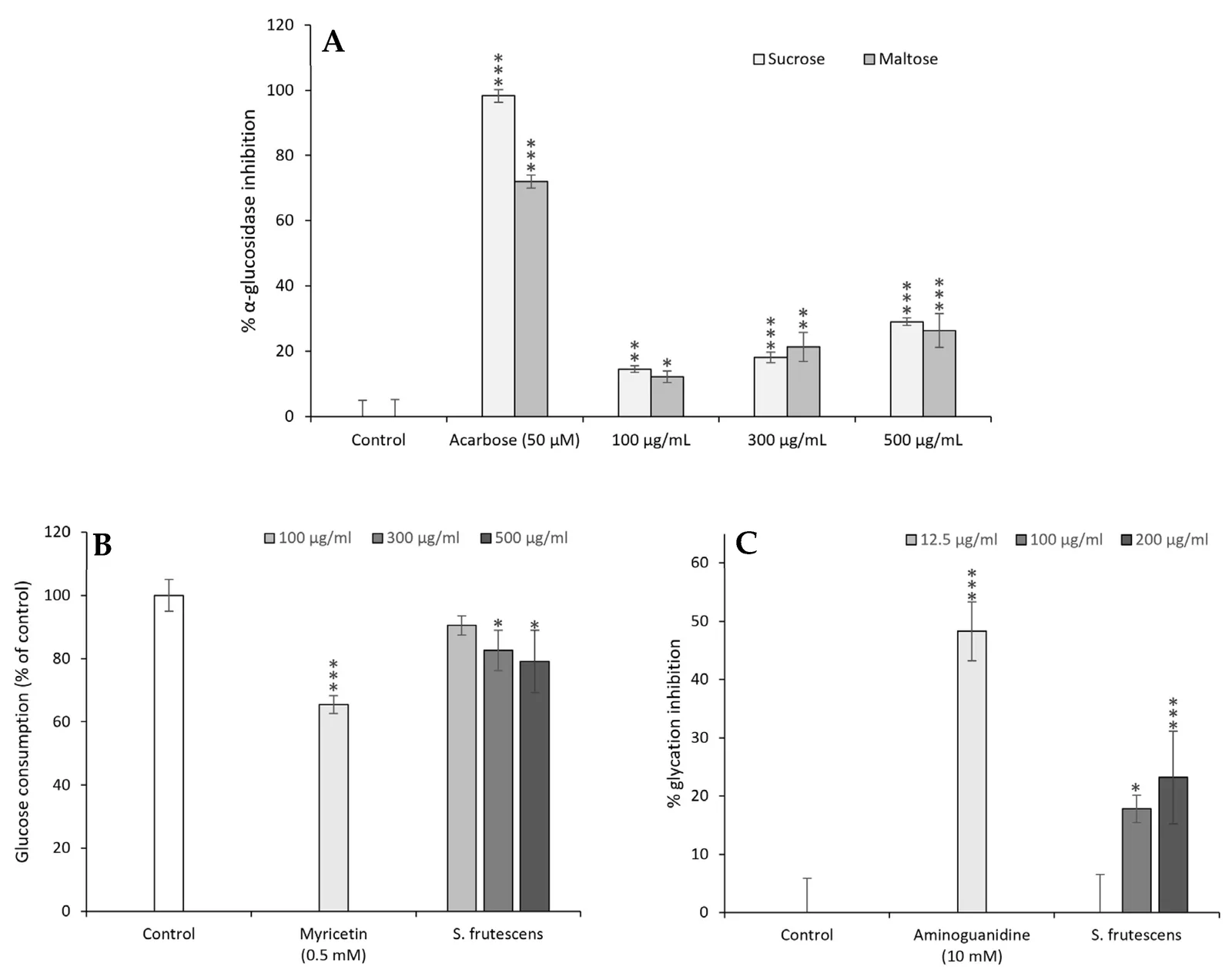
A comparison of (**A**) α-glucosidase inhibitory activities using maltose and sucrose as substrates with (**B**) the inhibition of intestinal glucose uptake in Caco-2 cells after 3 h for *Sutherlandia frutescens* hot aqueous extract. Acarbose is included as a positive control for α-glucosidase inhibition while myricetin (0.5 mM) is included as a positive control for Caco-2 glucose uptake. Glucose consumption is normalised for total cell numbers using Hoechst 33342 staining. (**C**) The protein glycation inhibitory potential of *S. frutescens* is also measured using glycated BSA with aminoguanidine included as a positive control. The results are reported as the mean ± standard deviation (error bars shown in black) with all experiments performed three times, each in triplicate (ANOVA and Tukey HSD post hoc test—* *p* < 0.05; ** *p* < 0.01; and *** *p* < 0.005—compared to control).

Another strategy for combatting postprandial hyperglycaemia is to reduce the absorption of glucose across the enterocyte brush border and into the blood stream. Accordingly, the ability of the aqueous extract of *S. frutescens* to combat postprandial hyperglycaemia by targeting carbohydrate digestion was explored by measuring its α-amylase and α-glucosidase inhibitory potentials using an enzyme-based approach. The ability of *S. frutescens* to reduce intestinal glucose absorption was also assessed by monitoring intestinal glucose uptake in a Caco-2 cell model (Figure 3B).

While *S. frutescens* was not shown to meaningfully inhibit the activity of α-amylase up to a concentration of 500 µg/mL (Figure S2, Supplementary Material), it demonstrated promising intestinal α-glucosidase inhibitory potential (Figure 3A) when using maltose or sucrose as substrates, in addition to a concentration-dependent decrease in intestinal glucose absorption (Figure 3B). To date, no direct evidence confirming the α-glucosidase inhibitory effects of *S. frutescens* could be found. While the study conducted by [20] demonstrated an inhibitory effect on intestinal glucose uptake in pre-diabetic male Wistar rats treated with *S. frutescens,* demonstrating a more potent effect than the oral antihyperglycemic agent metformin, it did not evaluate the direct effects of *S. frutescens* on glucose uptake as the rats were treated with the extract for a few weeks, after which intestinal glucose uptake was assessed in the absence of the extract. Consequently, the therapeutic effect described by [20] is distinct from the activities demonstrated in the present study and may represent distinct mechanisms that warrant future investigation in combination.

Due to the positive effects of DPP-IV inhibitors for reducing the severity of postprandial hyperglycaemia, the presence of natural DPP-IV inhibitors in *S. frutescens* was explored. *S. frutescens* resulted in a small decrease in the activity of the DPP-IV enzyme of 12.6% at 100 µg/mL (Supplementary Material Figure S3). This response is considered too weak to have a meaningful effect on its own, but may contribute to the overall multi-target pharmacological profile of the extract.

Postprandial hyperglycaemic spikes represent a major causal factor in the elevated AGE formation accompanying diabetic progression. Furthermore, advanced glycation end-products (AGEs) are known to exacerbate hyperglycaemia by hampering postprandial glucose disposal [45,46,47]. Inhibition of AGE formation represents a potential indirect therapeutic target to dampen postprandial hyperglycaemia and the subsequent progression to overt diabetes. Similar to the effects recorded for DPP-IV inhibition, the aqueous extract of *S. frutescens* weakly inhibited the glycation of BSA (Figure 3C), only reaching 23% inhibition at a concentration of 200 µg/mL (*p* < 0.05).

Collectively, the findings from the present study suggest that *S. frutescens* may influence multiple mechanisms involved in postprandial glucose regulation, an aspect that, to our knowledge, has not previously been comprehensively investigated in vitro or evaluated in an appropriate in vivo setting. This emphasises the value of target-directed in vitro screening for defining a more comprehensive mechanistic profile for medicinal plant-based therapeutics. Unlike current prescription drugs, such as acarbose, which only target carbohydrate digestion, *S. frutescens* may possibly modulate various inter-related mechanisms together, potentially contributing to improved glycaemic control.

### 3.4. Targets for Insulin Resistance and Inflammation

**Figure 4:**
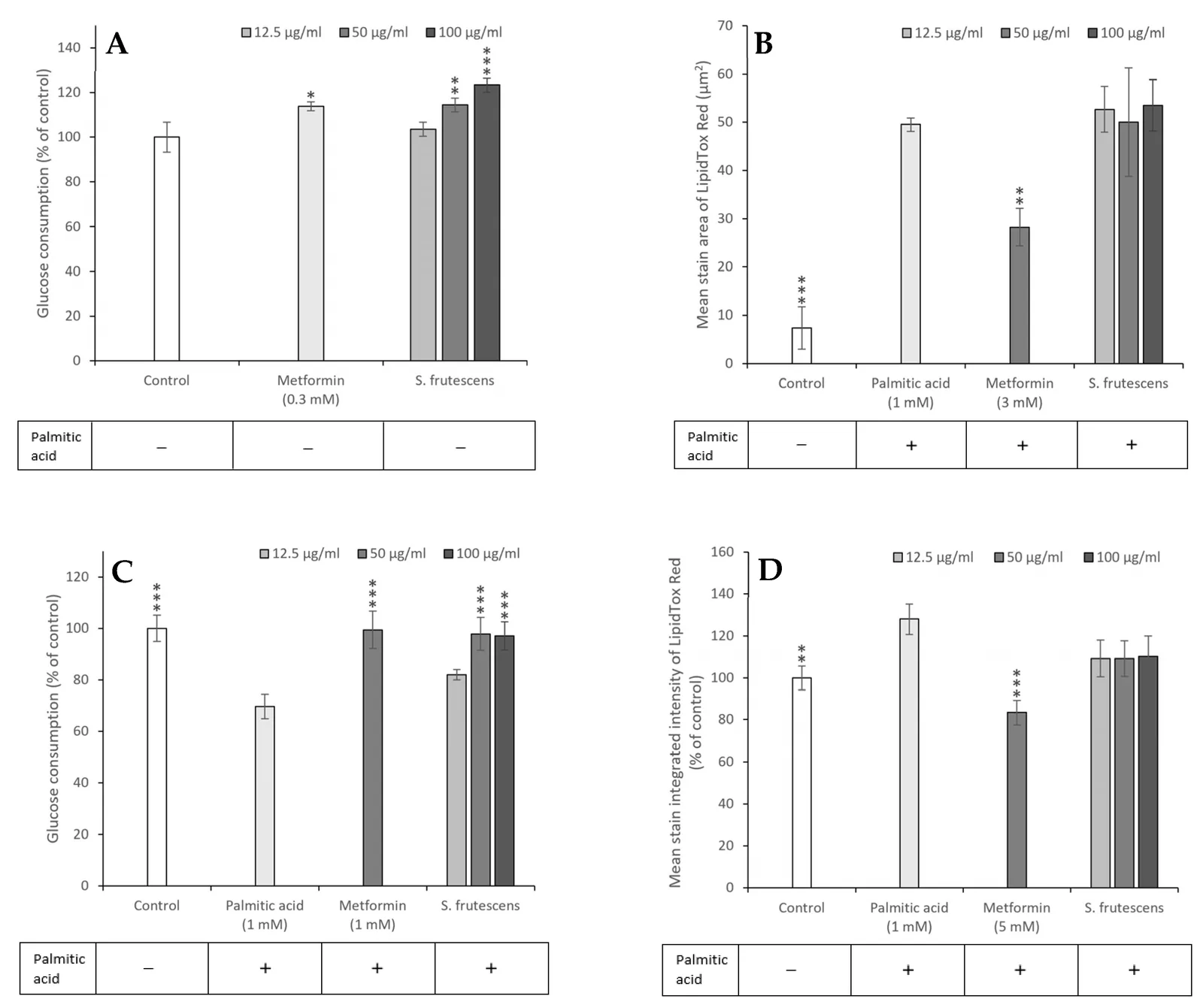
Glucose consumption and inhibition of lipid accumulation with *S. frutescens* hot aqueous extract, comparing (**A**) glucose consumption in C3A hepatocytes after 4 h following a 48 h treatment period, (**B**) lipid staining in C3A hepatocytes after a 24 h treatment period with palmitic acid, (**C**) glucose consumption in differentiated L6 skeletal myotubes after 3 h following a 24 h treatment period, and (**D**) lipid accumulation in differentiated L6 skeletal muscle cells in the presence of palmitic acid after 24 h. Metformin is included as a positive control to promote glucose uptake and prevent the palmitic acid-induced accumulation of lipids. All results are reported as the mean ± standard deviation (error bars shown in black) with all experiments performed three times, each in triplicate (ANOVA and Tukey HSD post hoc test—* *p* < 0.05; ** *p* < 0.01; and *** *p* < 0.005—compared to the control for (**A**) and compared to the palmitic acid control for (**B**–**D**)).

**Figure 5:**
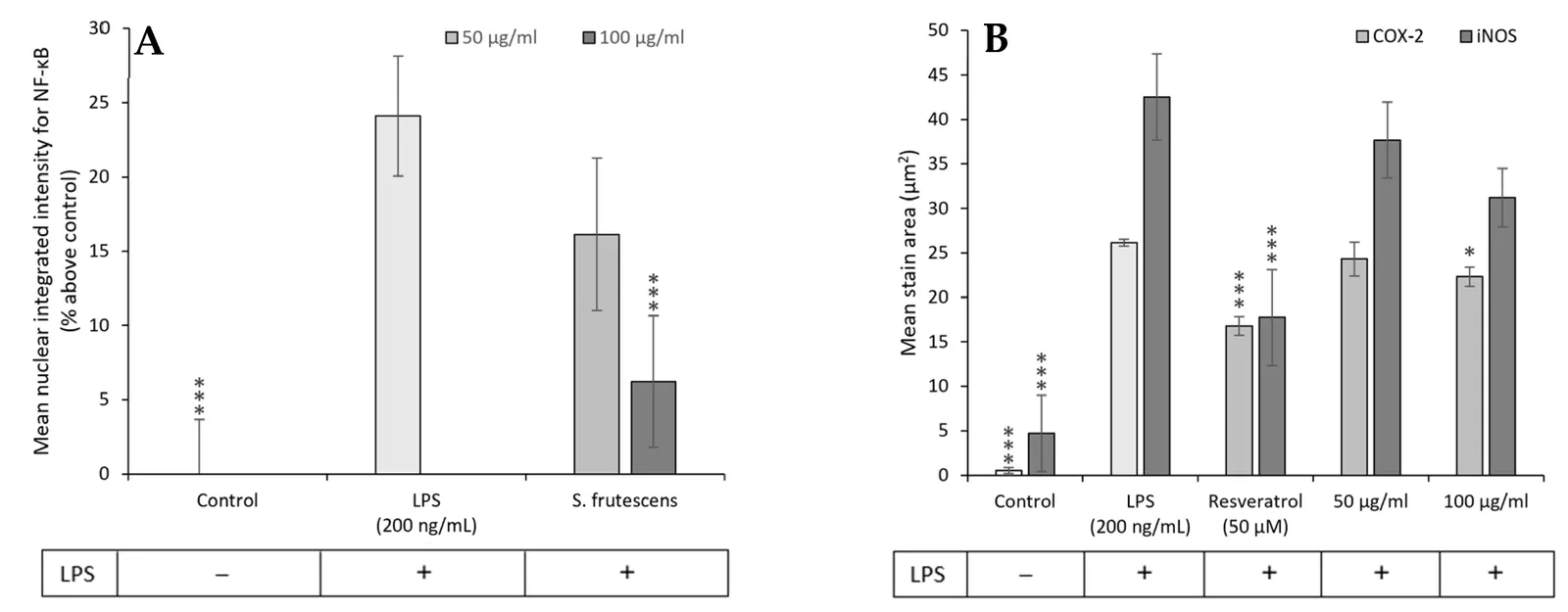
*S. frutescens* hot aqueous extract’s anti-inflammatory potential in RAW 264.7 cells activated with LPS, comparing (**A**) NF-κB nuclear translocation with (**B**) iNOS and COX-2 levels. Resveratrol is included as a positive control for the expression of iNOS and COX-2. Results are reported as the mean ± standard deviation (error bars shown in black) with all experiments performed three times, each in triplicate (ANOVA and Tukey HSD post hoc test—* *p* < 0.05; and *** *p* < 0.005—compared to LPS).

The excessive consumption of saturated fatty acids has long been identified as a causative agent of ectopic lipid accumulation, insulin resistance and the resultant development of T2D. Fatty acids may also initiate an inflammatory response by binding to the TLR4 receptor and subsequently activate the NF-κB pathway, leading to the production of inflammatory cytokines and impaired insulin sensitivity [48]. Accordingly, it is well accepted that altered glucose and lipid metabolism are prominent factors responsible for impaired insulin signalling [49], and thus represent potential therapeutic targets in the treatment of diabetes. Therefore, we evaluated the ability of S. frutescens to impact glucose metabolism in hepatocyte and myoblast models (Figure 4), as well as the NF-κB signalling pathway in macrophages (Figure 5).

The aqueous extract of *S. frutescens* successfully stimulated C3A glucose utilisation from 50 μg/mL (Figure 4A), and demonstrated a stronger capacity to stimulate glucose uptake at a concentration of 100 µg/mL compared to the positive control metformin (0.3 mM). *S. frutescens* did not, however, produce any notable effects on FFA-induced lipid accumulation up to a concentration of 100 µg/mL (Figure 4B). This appears to be in contrast to previous in vivo studies, which demonstrated a significant reduction in hepatic triglyceride content [12]; however, this is possibly an indirect effect due to the reduced serum FFAs in this in vivo HFD model, as suggested by [21]. In a different experimental setting, using fructose and insulin to promote insulin resistance in Chang liver cells, a crude hot water extract of *S. frutescens* was shown to ameliorate the effects of insulin resistance on lipid accumulation at concentrations as low as 12.5 μg/mL [10]. Although current sources of Chang liver cells may not be true representatives of mature hepatocytes, but rather a dedifferentiated derivative of a cervical cancer cell line [50], triglyceride accumulation occurred under experimental conditions of normal non-pathological FFA levels and is thus different from our C3A model exposed to excessive levels of palmitic acid. Therefore, the evidence suggests that *S. frutescens* does not impact directly on the capacity of hepatocytes to store excess serum FFA, but may reduce cellular de novo lipid accumulation, as reported by [10].

Due to the fact that skeletal muscle insulin resistance has been identified as the initiating factor or, at the very least, one of the primary defects contributing to the pathophysiology of T2D, the ability of *S. frutescens* to restore normal glucose utilisation and reduce lipid accumulation in response to palmitic acid-induced insulin resistance was examined using differentiated L6 cells. *S. frutescens* almost completely ameliorated the effects of palmitic acid on skeletal muscle function as it promoted glucose uptake (Figure 4C) by almost 30% (*p* < 0.005) while simultaneously causing a small but statistically insignificant reduction in intracellular lipid accumulation (Figure 4D) by 17% at extract concentrations of 50 and 100 μg/mL.

Previous studies conducted on insulin-resistant male Wistar rats that were fed a high-fat diet revealed that an aqueous extract of *S. frutescens* promoted skeletal muscle glucose uptake, in agreement with our in vitro findings (Figure 4) [12,20]. Pinitol, one of the bioactive compounds isolated from *S. frutescens*, has been reported to stimulate GLUT-4 translocation to the plasma membrane [51], thereby improving glucose uptake and providing a potential mechanism through which *S. frutescens* could promote skeletal muscle glucose utilisation. However, since glucose utilisation is also stimulated in cell types not strongly expressing the GLUT-4 transporter (C3A, Chang liver/HeLa and 3T3-L1 preadipocytes), alternative or additional mechanisms need to be considered. Canavanine, a guanidinium compound like metformin, can, via imidazoline receptors, activate glucose uptake by a mechanism involving AMPK, thus offering an additional mechanism for improved glucose uptake [52].

Increasing evidence suggests that obesity, and subsequent adipose dysfunction, are related to T2D through the development of insulin resistance. Ref. [12] reported that *S. frutescens* increased glucose utilisation and decreased triglyceride accumulation in an adipose model (3T3-L1 cells), establishing an in vitro effect of *S. frutescens* on adipose tissue function. PPARγ is a prominent and much-pursued antidiabetic target aiming to restore insulin sensitivity. As a result, 3T3-L1 differentiation is now a popular screening tool to identify potential PPARγ activators. Using triglyceride accumulation and glycerol-3-phosphate dehydrogenase activity as markers for adipogenesis in 3T3-L1 cells, no significant adipogenic differentiation was evident for the *S. frutescens* extract (up to a concentration of 200 µg/mL) compared to that of the PPARγ agonist rosiglitazone [12]. However, *S. frutescens* was strongly antagonistic toward rosiglitazone-induced adipogenesis in this experimental setting. While the inability to stimulate adipogenesis in 3T3-L1 cells rules out a full PPARγ agonist mode of action, it is possible to consider *S. frutescens* as a partial agonist, as those typically enhance glucose uptake but do not stimulate triglyceride accumulation [53,54], a situation similar to that observed for *S. frutescens*.

At least two independent in vivo studies have indicated that *S. frutescens* treatment leads to significant weight loss in HFD-fed rats, supporting the in vitro 3T3-L1 differentiation data reported here [12,20]. Furthermore, ref. [12] also demonstrated a specific reduction in adipose tissue triglyceride levels, contrasting with the typical characteristics of full PPARγ agonists, such as rosiglitazone [54]. At this stage, however, we can not rule out the possibility that these effects are completely independent of PPARγ activation, making further studies necessary in order to clarify the mechanism.

Given that dietary triglycerides contribute to the development of obesity as well as heightened postprandial FFA levels, the capacity of *S. frutescens* to inhibit pancreatic lipase was measured. The aqueous extract of *S. frutescens* was not, however, found to inhibit this enzyme up to a concentration of 500 µg/mL (Supplementary Material, Figure S4).

In addition to the role of lipolysis and FFAs in the progression of obesity and related metabolic disturbances, adipose tissue also houses various immune cells, such as tissue-specific resident macrophages. In obesity, adipose tissue dysfunction is characterised by an increased inflammatory macrophage component; subsequently, T2D is defined by low-grade chronic inflammation [55]. To assess the anti-inflammatory potential of *S. frutescens*, NF-κB nuclear translocation and activation of associated downstream inflammatory markers, iNOS and COX2, were explored using LPS-activated RAW 264.7 macrophage-like cells as a model system (Figure 5). *S. frutescens* reduced the nuclear accumulation of NF-κB by 18% (Figure 5A), providing evidence to support a potential anti-inflammatory response. Downstream responders, iNOS and COX2 protein levels were also reduced at an extract concentration of 100 μg/mL, although the reduction was only significant for COX2 (*p* = 0.067 and *p* < 0.05, respectively). Ref. [17] reported a similar inhibition in NF-ĸB nuclear translocation in response to *S. frutescens*, as well as decreased expression of both iNOS and COX2.

A previous study reported that an ethanolic extract of *S. frutescens* reduced NO production and iNOS expression in murine macrophages activated by LPS and IFN-γ in a dose-dependent manner at extract concentrations ranging from 50 to 200 μg/mL. Interestingly, this activity did not appear to be mediated by sutherlandiosides or sutherlandins; however, chlorophyll appeared to be partially responsible for these effects [16]. As an aqueous extract, one may anticipate that the extract used in the present study is essentially chlorophyll free.

### 3.5. Pancreatic Beta-Cell Function

**Figure 6:**
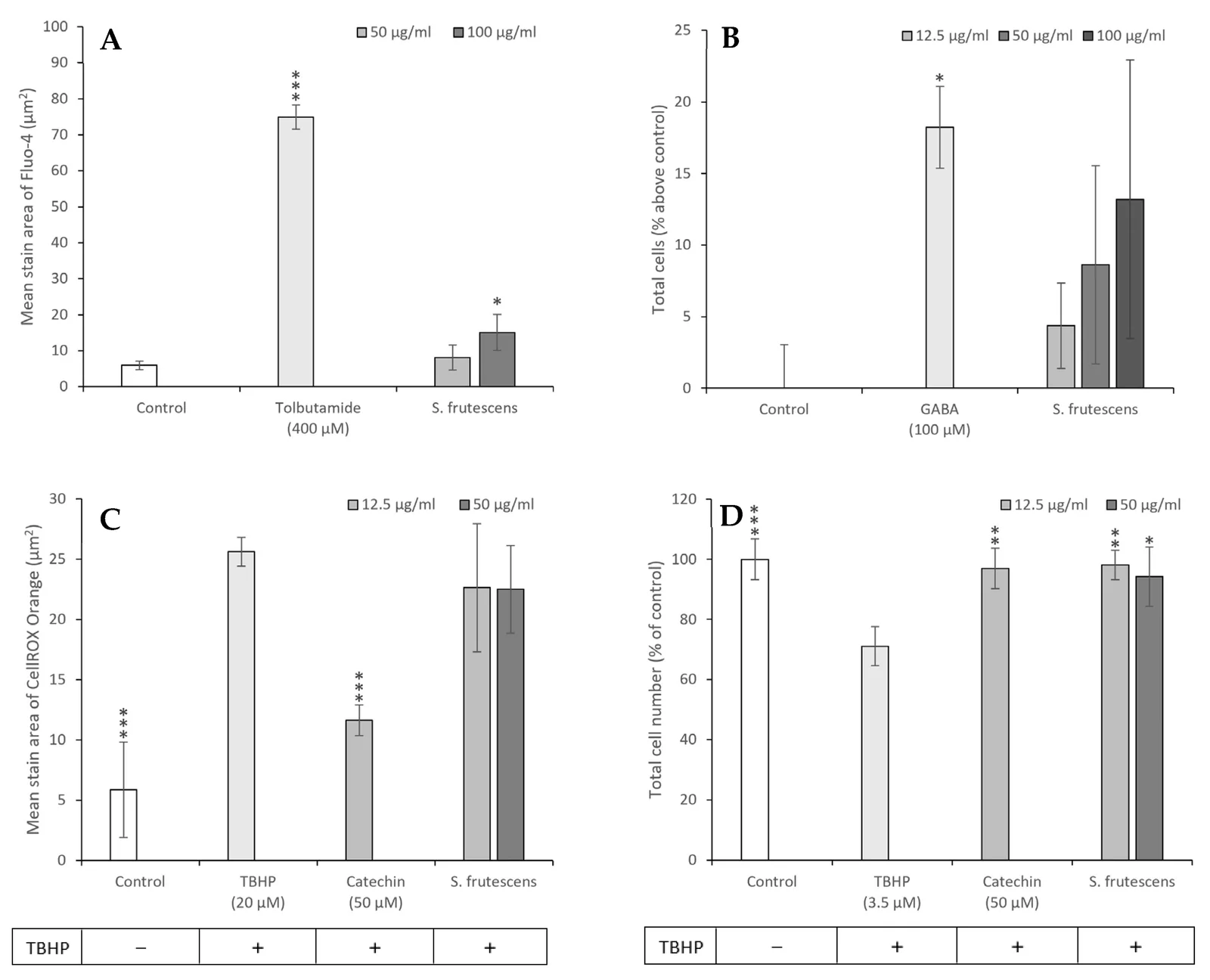
Function and survival of INS-1 pancreatic β-cells in the presence of *S. frutescens* hot aqueous extract, comparing (**A**) intracellular calcium levels in the absence of glucose after a 10 min treatment period with (**B**) β-cell proliferation after a 24 h incubation period measured using Hoechst 33342 staining of the nuclei, (**C**) intracellular ROS accumulation following exposure to TBHP for 2 h measured using the CellROX Orange reagent, and (**D**) promotion of cell survival in the presence of TBHP to promote oxidative stress-induced cell death after a 24 h treatment period. Tolbutamide and gamma-aminobutyric acid (GABA) are included as positive controls for calcium influx and β-cell proliferation, respectively. Results are reported as the mean ± standard deviation (error bars shown in black) with all experiments performed three times, each in triplicate (ANOVA and Tukey HSD post hoc test—* *p* < 0.05; ** *p* < 0.01; and *** *p* < 0.005—compared to control for (**A**,**B**) and compared to TBHP for (**C**,**D**)).

Chronically elevated blood glucose levels have detrimental consequences on insulin synthesis and secretion, β-cell survival, and insulin sensitivity; thus, treatment strategies that promote β-cell mass and function could offer unparalleled advantages in patients with T1D or T2D [56]. Subsequently, the effects of *S. frutescens* on insulin secretion, oxidative stress, cell proliferation and cell survival were assessed in INS-1 pancreatic β-cells (Figure 6).

The aqueous extract of *S. frutescens* produced a mild but statistically significant effect on intracellular calcium flux in the absence of glucose by producing a 2.5-fold increase (*p* = 0.05) at the highest tested extract concentration of 100 μg/mL compared to the control (Figure 6A). These results confirm those from a previous study, which showed that this plant species promotes calcium influx and measured insulin secretion in INS-1 cells in a dose-dependent manner between 25 and 100 μg/mL [11].

Considering that *S. frutescens* has been reported to contain significant quantities of GABA [7,56,57], and that a substantial literature demonstrates GABA as a stimulator of β-cell proliferation both in vitro and in vivo [13,14,58], we tested the impact of *S. frutescens* on INS-1 proliferation. However, no statistically significant proliferatory effect could be demonstrated based on the differences in cell numbers (Figure 6B).

Pancreatic β-cells are unique in their extreme vulnerability to oxidative stress [59]. During the present study, the aqueous extract of *S. frutescens* presented relatively weak antioxidant effects as defined by the FRAP (10.31 Trolox equivalents (g/g)) and DPPH scavenging (EC50: 45.58 µg/mL) assays and, in line with this poor antioxidant capacity, could not attenuate ROS levels in TBHP-challenged INS-1 β-cells (Figure 6C). Although a number of previous reports have claimed *S. frutescens* is a promising adjuvant for health problems related to oxidative stress, alternative in vitro assays [15,60] and excessive extract concentrations [61] were used to demonstrate such activity. While it has been previously suggested that *S. frutescens* may reduce the effects of NO and other ROS on β-cells due to the presence of L-canavanine and pinitol compounds [7,8,62], no studies documenting the antioxidant effects of *S. frutescens* on β-cells—a unique cellular setting with regard to ROS production and inherent antioxidant mechanisms—could be found. Despite its apparently weak antioxidant capacity, *S. frutescens* significantly (*p* < 0.01) promoted cell survival, almost completely ameliorating the cytotoxic effects of ROS on INS-1 cells at an extract concentration of 12.5 μg/mL (Figure 6D). This indicates that while *S. frutescens* extract has little capacity to directly counteract ROS levels, it can effectively enhance cellular resistance to oxidative stress and protect β-cell viability.

The heighted vulnerability of pancreatic β-cells to oxidative stress has long been exploited in developing animal models for diabetes. Both alloxan and streptozotocin decimate β-cells through mechanisms involving oxidative stress [63], resulting in insulin insufficiency and the concomitant development of hyperglycaemia. The study by [19], which utilised streptozotocin to induce diabetes, provides the only in vivo evaluation of *S. frutescens* in a model representing β-cell destruction. As detailed above, our in vitro data suggests that increased insulin secretion and β-cell resistance to both oxidative stress and cell death represent potential mechanisms that may have contributed to the improved glycaemic control noted in the study by [19]. Canavanine, a bioactive component of *S. frutescens* and an agonist to imidazoline receptors, has been shown to reduce hyperglycaemia in STZ-induced diabetic rats in a receptor-dependent manner [64]. Furthermore, activation of the imidazoline I-3 receptor increases calcium levels and subsequent insulin secretion in pancreatic β-cells both in vitro and in vivo [65]. Receptor activation by various ligands has also shown the capacity to protect β-cells from damage induced by STZ (reviewed by [66]); however the precise mechanism has not been elucidated. Collectively, these activities of canavanine overlap remarkably well with the in vitro effects of *S. frutescens* extract on INS-1 β-cells.

A summary table of all the in vitro bioactivity results is available as Table S1 in the Supplementary Materials.

## 4. Discussion

A small number of publications have previously documented the potential antidiabetic effects of *S. frutescens*, claiming the presence of various active ingredients likely responsible for its antidiabetic activity, including pinitol, GABA, canavanine and a series of cycloartane-type triterpenoid saponins [7,8]. With the exception of SU1, the main cycloartane-type triterpenoid found in *S. frutescens*, and a compound which remains therapeutically uncharacterised to date, many of the in vitro mechanisms identified in this study can indeed be related to the reported biological properties of these known active ingredients. It is also noteworthy that cycloartane triterpenoids are themselves a well-studied group of compounds and have been reported to possess diverse antidiabetic activities [67,68].

To date, *S. frutescens* has been investigated in two distinct animal models addressing different aspects of glucose dysregulation, including hyperglycaemia associated with β-cell atrophy and insulin resistance induced by high-fat feeding. Although these studies provide evidence of biological activity under specific experimental conditions, they do not address the broader range of mechanisms that may contribute to the effects of this complex plant extract. Our in vitro findings are consistent with several observations from these earlier in vivo studies while also identifying additional biological activities that, to our knowledge, have not previously been characterised in *S. frutescens*. As an example, *S. frutescens* demonstrated the potential to inhibit multiple targets relevant to postprandial hyperglycaemia, as supported by the statistically significant responses towards carbohydrate digestion, intestinal glucose uptake in Caco-2 cells, and protein glycation. Controlling postprandial hyperglycaemic spikes is a recognised therapeutic strategy, particularly during the early phase of diabetes development [69,70].

To the best of our knowledge, no in vivo studies evaluating the potential effects of *S. frutescens* on postprandial hyperglycaemia have been reported to date. Furthermore, interventions aimed at reducing postprandial hyperglycaemia are most effective when administered in direct temporal association with the glycaemic load, a condition that has not been adequately addressed in the published in vivo studies on *S. frutescens*. The present findings suggest that the extract influences several mechanisms relevant to postprandial glucose regulation, including delayed carbohydrate digestion and reduced intestinal glucose uptake. In addition, the observed effects on β-cell function and glucose uptake in adipose and muscle cells may represent complementary mechanisms that could influence postprandial glucose handling, given the central role of insulin-sensitive tissues in postprandial glucose disposal. However, whether these combined activities translate into meaningful reductions in postprandial hyperglycaemia in vivo remains to be established.

Obesity is now considered a prominent component driving the emerging diabetes pandemic. It thus follows that targeting adipose function is a highly relevant antidiabetic strategy. Using a HFD model, MacKenzie and co-workers provided evidence demonstrating *S. frutescens* to have a strong lipocentric antidiabetic mechanism. An improved serum lipid profile and a reduction in adipose mass, triglyceride content, and animal weight support a therapeutic role involving adipose tissue function [12]. In an earlier study, however, these authors demonstrated that improvements in the serum lipid profile manifested prior to insulin resistance in this diabetic model, raising the likelihood that the reported antidiabetic activity was independent of obesity-induced adipose tissue dysfunction, the primary hallmark of human T2D [21]. Furthermore, relative to the LFD control group, and despite a clear increase in body weight, this HFD model did not reveal any characteristic changes in the serum cytokine profile, thereby providing additional evidence that their model is essentially independent of obesity [21]. Therefore, it remains to be established what beneficial effects *S. frutescens* may have in a model more closely mimicking the contribution of obesity to the development and treatment of human diabetes.

Similarly, although the anti-inflammatory potential of *S. frutescens* has been evaluated in various animal models [19,71], there has been no direct in vivo indication as to the impact of this anti-inflammatory activity in a diabetic model representative of obesity-driven adipose tissue inflammation. With NF-κB as the central hub coordinating divergent inflammatory responses, it is not surprising that several *S. frutescens* studies, including the present one, indicate anti-inflammatory activity through an attenuated NF-ĸB signalling mechanism [17,72]. Taken together, substantial in vitro and in vivo evidence now exists to warrant a detailed study on the impact of *S. frutescens* in a model representative of obesity-driven diabetes. The current evidence suggests the reported lipocentric mechanism may be novel in that it does not involve a typical PPARγ agonist response, a therapeutic target extensively pursued in the past for drug development and often dismissed due to toxicity and unwanted weight gain.

In light of the potential protective effects of *S. frutescens* on pancreatic β-cells revealed in the present study, investigating whether *S. frutescens* could delay the onset of type 1 diabetes presents another interesting research avenue for this plant species that has not been adequately explored. Both canavanine and GABA, through receptors confirmed to be present on pancreatic β-cells, have been demonstrated to improve β-cell function and mass [58,62,73]. These findings highlight the need for well-designed in vivo studies to validate the mechanistic effects observed in the present study and to determine their physiological relevance. Such studies would provide a stronger foundation for assessing the therapeutic potential of *S. frutescens* and informing future preclinical development.

The interpretation of individual target responses should also take into account the pharmacological complexity of multicomponent botanical extracts. Whereas conventional antidiabetic agents were generally developed to achieve potent activity against a defined molecular target, complex botanical extracts may act through the simultaneous modulation of multiple biological pathways, consistent with the principles of network pharmacology [74,75]. Consequently, modest activity at an individual target should not necessarily be interpreted in isolation, particularly when several complementary mechanisms are affected. Previous studies of *S. frutescens* provide some support for this interpretation, with relatively modest in vitro activities occurring alongside reported effects on insulin resistance and glycaemic control in animal models [19,20,21,62]. This distributed, mild multi-target activity mirrors patterns seen in other complex plant extracts, where weak in vitro inhibition still yields potent systemic glucose regulation in vivo [76,77]. Importantly, this interpretation does not imply that the individual activities demonstrated here are independently sufficient to produce a therapeutic effect, nor does it establish additive or synergistic interactions. Rather, the findings support the possibility that the biological effects of a complex extract may emerge from the coordinated modulation of multiple interrelated pathways, representing a pharmacological strategy distinct from that of conventional single-target agents.

**Figure 7:**
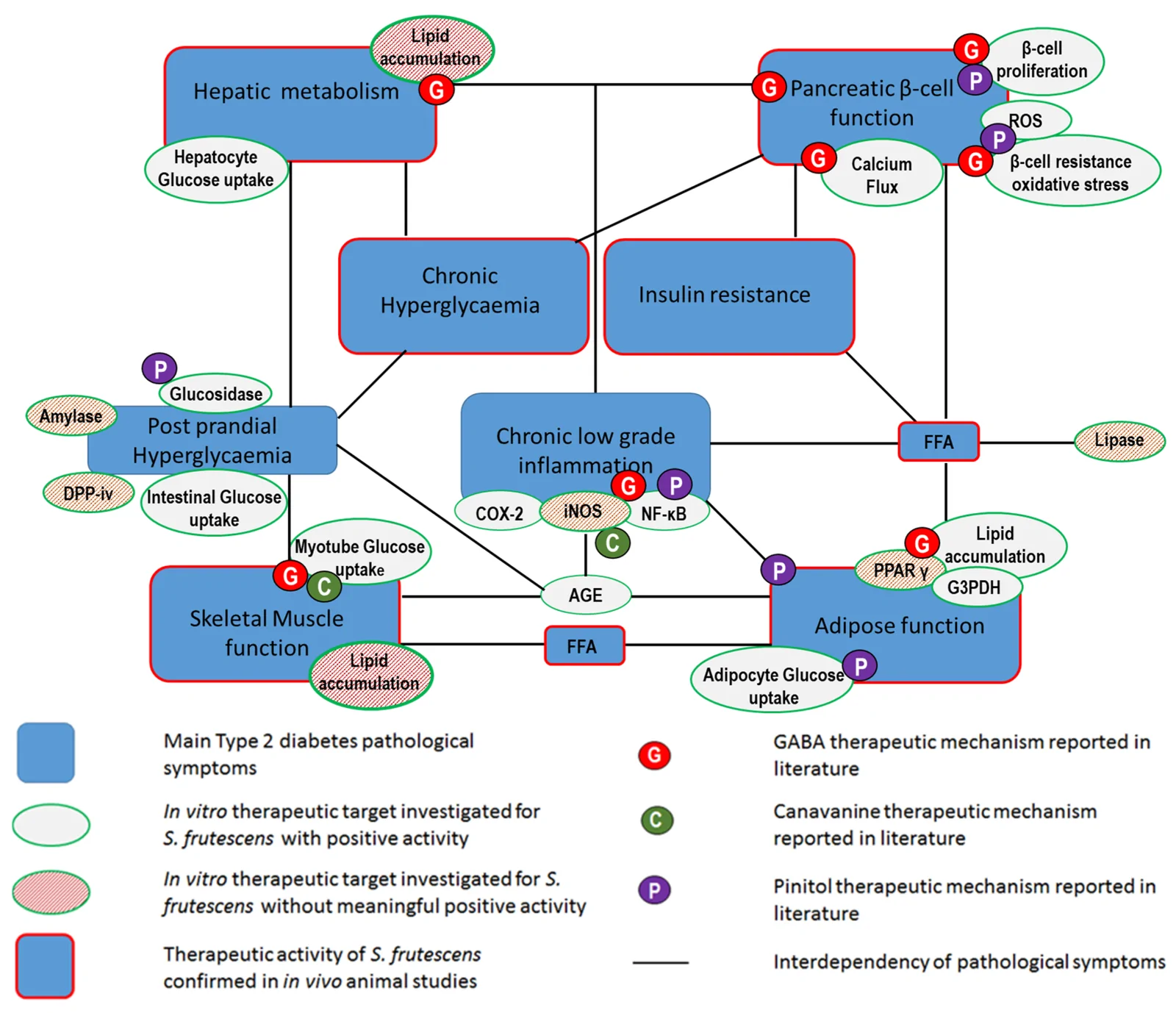
Integrated multi-target activity of *S. frutescens* extract. Illustration highlighting the main type 2 diabetic pathological symptoms, their interdependency, and the potential therapeutic activities identified through target-directed in vitro screening, overlaid with previously published animal studies and reported biological activities of key compounds.

The integrated nature of the present screening platform enables the mechanistic findings to be viewed collectively rather than as isolated observations. Figure 7 presents a conceptual overview of the principal biological activities identified in this study, together with their relationship to previously reported in vivo observations. This integrated framework illustrates how comprehensive target-directed in vitro screening can complement animal studies by identifying the predominant mechanisms of action and informing the selection of appropriate models for subsequent in vivo evaluation.

## 5. Conclusions

Overall, our data highlight *S. frutescens* as a multicomponent therapeutic with the capacity to engage numerous antidiabetic targets collectively, thus emphasising the need to view this medicinal plant differently from conventional drugs, for which the focus has been to develop potent singular mode of action treatments. Such a paradigm shift requires innovative strategies to accurately define the anticipated complex pharmacology of more subtle multi-target therapeutic activities, especially in the context of diabetes, where animal models have significant limitations in their ability to mimic the full complement of the human disease. In the present study, we explored target-directed in vitro screening as a potential approach to complement antidiabetic animal models. The high-throughput capacity of in vitro assays, together with modern technological advances in cellular phenotypic data analysis (cellomics), provide a diverse yet practical research setting for the comprehensive characterisation of the biological activities of complex, multicomponent plant extracts. Rather than replacing in vivo studies, such an approach can guide the selection of appropriate animal models for subsequent investigation by identifying the predominant mechanisms of action and informing the choice of models, in which factors such as absorption, metabolism and bioavailability can be evaluated.

As expected for a comprehensive screening study comprising multiple independent cell-based assays, some endpoints exhibited greater biological variability than others. Although this variability reduced statistical power for certain measurements, the direction of the responses remained consistent across independent experiments, supporting the overall mechanistic trends observed. Nevertheless, confirmation of these findings in targeted follow-up studies with larger sample sizes would further strengthen the conclusions.

A further limitation of the present study is that the biological activities were evaluated using a crude aqueous extract. Consequently, the relative contributions of individual constituents to the observed biological activities could not be established, nor could potential additive or synergistic interactions between the constituents be distinguished. While such studies would provide valuable insight into the phytochemical basis of the observed activities, it is also recognised that the pharmacology of medicinal plant extracts may emerge from the integrated actions of multiple constituents rather than from a single dominant active compound.

While we have made a concerted effort to include a wide representation of current recognised antidiabetic targets, it is acknowledged that our antidiabetic screening platform is by no means exhaustive. Notwithstanding, in vitro target-directed screening reveals a greater complexity than previously acknowledged and underscores the potential of *S. frutescens* as a multicomponent antidiabetic therapy, distinct from mainstream drug development objectives, which place absolute emphasis on the identification of individual active ingredients. Using an identical in vitro approach, we recently demonstrated a similar mechanistic complexity for extracts prepared from the antidiabetic plant *Aspalathus linearis*, further supporting the value of target-directed in vitro screening as a complementary approach toward improving the accuracy of defining the pre-clinical antidiabetic properties of medicinal plants [4].

## Data Availability

The original contributions presented in this study are included in the Supplementary Material. Further inquiries can be directed to the corresponding author.

## Supplementary Materials

The following supporting information can be downloaded at: https://www.mdpi.com/article/10.3390/life16081348/s1. Figure S1: Basal cytotoxicity screening of *S. frutescens* hot aqueous extract comparing cells stained positive for propidium iodide (PI) staining for C3A hepatocytes after 48 h, differentiated L6 myotubes after 24 h, RAW 264.7 macrophages in the presence of LPS after 48 h, INS-1 pancreatic β-cells after 24 h and Caco-2 cells after 3 h. Figure S2: The α-amylase inhibitory activities of *S. frutescens*. Acarbose was included as a positive control. Figure S3: The DPP-IV inhibitory activity of *S. frutescens*. Figure S4: The pancreatic lipase inhibitory activities of *S. frutescens*. TABLE S1: Summary of the in vitro screening assays and the respective outcomes (Pringle et al.).

## Abbreviations

The following abbreviations are used in this manuscript:

AGE	Advanced glycation end-product
Akt	Protein kinase B
AMPK	5′ adenosine monophosphate-activated protein kinase
BEH	Ethylene-bridged hybrid
BSA	Bovine serum albumin
Ca^2+^	Calcium ion
CO_2_	Carbon dioxide
COX	Cyclooxygenase
DMEM	Dulbecco’s Modified Eagle Medium
DMSO	Dimethyl sulfoxide
DPPH	2,2-diphenyl-1-picrylhydrazyl
DPP-IV	Dipeptidyl peptidase-iv
EDTA	Ethylenediaminetetraacetic acid
FBS	Foetal bovine serum
FFA	Free fatty acids
FRAP	Ferric reducing antioxidant power
GABA	γ-aminobutyric acid
GLUT	Glucose transporter
Gly-Pro pNA	Gly-Pro p-nitroanilide hydrochloride
HCl	Hydrochloric acid
HFD	High-fat diet
IFN	Interferon
iNOS	Inducible nitric oxide synthase
KCl	Potassium chloride
KH_2_PO_4_	Potassium dihydrogen phosphate
LFD	Low-fat diet
LPS	Lipopolysaccharide
MEM	Minimum essential medium
Mg^2+^	Magnesium ion
N_2_	Nitrogen
Na_2_HPO_4_	Disodium phosphate
NaCl	Sodium chloride
NEAAs	Non-essential amino acids
NF-κB	Nuclear factor kappa B
NO	Nitric oxide
PBS	Phosphate-buffered saline
PDA	Photodiode array
PE	Phycoerythrin
PI	Propidium iodide
PI3K	Phosphoinositide 3-kinase
pNPP	p-nitrophenyl palmitate
PPAR	Peroxisome proliferator-activated receptor
ROS	Reactive oxygen species
RPMI	Roswell Park Memorial Institute
STZ	Streptozotocin
SU1	Sutherlandioside B
T1D	Type 1 diabetes
T2D	Type 2 diabetes
TBHP	tert-Butyl hydroperoxide
TE	Trolox equivalents
TLR	Toll-like receptor
TPP	2,3,5-triphenyl-1,3,4-triaza-2-azoniacyclopenta1,4-diene chloride
TPTZ	2,4,6-tris(2-pyridyl)-1,3,5-triazine
UPLC-MS	Ultra-performance liquid chromatography–mass spectrometry
WFI	Water for injection

## References

[1] Guo H., Wu H., Li Z. (2023). The pathogenesis of diabetes. Int. J. Mol. Sci..

[2] Yameny A.A. (2025). Diabetes mellitus: A comprehensive review of types, pathophysiology, complications, and standards of care in diabetes. J. Med. Life Sci..

[3] Mohammed A., Ibrahim M.A., Islam M.S. (2014). African medicinal plants with antidiabetic potentials: A review. Planta Medica.

[4] Pringle N.A., van de Venter M., Koekemoer T. (2021). Comprehensive in vitro antidiabetic screening of *Aspalathus linearis* using a target-directed screening platform and cellomics. Food Funct..

[5] Van Wyk B.-E., van Oudtshoorn B., Gericke N. (2009). Medicinal Plants of South Africa.

[6] Mncwangi N., Viljoen A., Mulaudzi N., Fouche G. (2023). *Lessertia* *frutescens*. The South African Herbal Pharmacopoeia.

[7] Van Wyk B.-E., Albrecht C. (2008). A review of the taxonomy, ethnobotany, chemistry and pharmacology of *Sutherlandia frutescens* (Fabaceae). J. Ethnopharmacol..

[8] Aboyade O.M., Styger G., Gibson D., Hughes G. (2014). *Sutherlandia frutescens*: The meeting of science and traditional knowledge. J. Altern. Complement. Med..

[9] Ndjoubi K.O., Sharma R., Hussein A.A. (2025). Phytochemistry, ethnopharmacology, and pharmacology of *Lessertia frutescens* (Cancer Bush): A comprehensive review. Plants.

[10] Williams S., Roux S., Koekemoer T., van de Venter M., Dealtry G. (2013). *Sutherlandia frutescens* prevents changes in diabetes-related gene expression in a fructose-induced insulin resistant cell model. J. Ethnopharmacol..

[11] Elliot G.P. (2011). Implementation of Novel Flow Cytometric Methods to Assess the In Vitro Antidiabetic Mechanism of a *Sutherlandia frutescens* Extract. Ph.D. Thesis.

[12] MacKenzie J., Koekemoer T.C., Roux S., van de Venter M., Dealtry G.B. (2012). Effect of *Sutherlandia frutescens* on the lipid metabolism in an insulin resistant rat model and 3T3-L1 adipocytes. Phytother. Res..

[13] Liu W., Jin T., Wang Q. (2019). 337-LB: Combined Oral Administration of GABA and GLP-1 Promotes Human ß-Cell Proliferation and Reduces Apoptosis. Diabetes.

[14] Soltani N., Qiu H., Aleksic M., Glinka Y., Zhao F., Liu R., Li Y., Zhang N., Chakrabarti R., Ng T. (2011). GABA exerts protective and regenerative effects on islet beta cells and reverses diabetes. Proc. Natl. Acad. Sci. USA.

[15] Fernandes A.C., Cromarty A.D., Albrecht C., van Rensburg C.E. (2004). The antioxidant potential of *Sutherlandia frutescens*. J. Ethnopharmacol..

[16] Lei W., Browning J.D., Eichen P.A., Brownstein K.J., Folk W.R., Sun G.Y., Lubahn D.B., Rottinghaus G.E., Fritsche K.L. (2015). Unveiling the anti-inflammatory activity of *Sutherlandia frutescens* using murine macrophages. Int. Immunopharmacol..

[17] Camille N., Dealtry G. (2018). Regulation of M1/M2 macrophage polarization by *Sutherlandia frutescens* via NFkB and MAPK signaling pathways. S. Afr. J. Bot..

[18] Fortuin-Seedat M., Camille N., Adefuye J., Oosthuysen M., Dealtry G.B. (2018). Does Sutherlandia frutescens influence the immune system via regulation of macrophage polarization and function?. Appl. Cell Biol..

[19] Ojewole J.A. (2004). Analgesic, antiinflammatory and hypoglycemic effects of *Sutherlandia frutescens* R. BR. (variety Incana E. MEY.) [Fabaceae] shoot aqueous extract. Methods Find. Exp. Clin. Pharmacol..

[20] Chadwick W.A., Roux S., van de Venter M., Louw J., Oelofsen W. (2007). Anti-diabetic effects of *Sutherlandia frutescens* in Wistar rats fed a diabetogenic diet. J. Ethnopharmacol..

[21] MacKenzie J., Koekemoer T., van de Venter M., Dealtry G., Roux S. (2009). *Sutherlandia frutescens* Limits the Development of Insulin Resistance by Decreasing Plasma Free Fatty Acid Levels. Phytother. Res..

[22] Eddouks M., Chattopadhyay D., Zeggwagh N.A. (2012). Animal models as tools to investigate antidiabetic and anti-inflammatory plants. Evid. Based Complement. Altern. Med..

[23] Heydemann A. (2016). An Overview of Murine High Fat Diet as a Model for Type 2 Diabetes Mellitus. J. Diabetes Res..

[24] Chessel A., Carazo Salas R.E. (2019). From observing to predicting single-cell structure and function with high-throughput/high-content microscopy. Essays Biochem..

[25] Mennacozzi M., Landesmann B., Harris G.A., Liska R., Whelan M. (2012). Hepatotoxicity screening taking a mode-of-action approach using HepaRG cells and HCA. Altex Proc..

[26] Donato M.T., Tolosa L. (2021). High-content screening for the determination of drug-induced oxidative stress in liver cells. Antioxidants.

[27] Mavimbela T., Vermaak I., Chen W., Viljoen A. (2018). Rapid quality control of *Sutherlandia frutescens* leaf material through the quantification of SU1 using vibrational spectroscopy in conjunction with chemometric data analysis. Phytochem. Lett..

[28] Xiao Z., Storms R., Tsang A. (2006). A quantitative starch–iodine method for measuring alphaamylase and glucoamylase activities. Anal. Biochem..

[29] Oki T., Matsui T., Osajima Y. (1999). Inhibitory effect of alpha-glucosidase inhibitors varies according to its origin. J. Agric. Food Chem..

[30] Lott J.A., Turner K. (1975). Evaluation of Trinder’s glucose oxidase method for measuring glucose in serum and urine. Clin. Chem..

[31] Kwon O., Eck P., Chen S., Corpe C.P., Lee J.-H., Kruhlak M., Levine M. (2007). Inhibition of the intestinal glucose transporter GLUT2 by flavonoids. FASEB J..

[32] Matsuura N., Aradate T., Sasaki C., Kojima H., Ohara M., Hasegawa J., Ubukata M. (2002). Screening system for the Maillard reaction inhibitor from natural product extracts. J. Health Sci..

[33] Boukes G.J., van de Venter M. (2012). Rooperol as an antioxidant and its role in the innate immune system: An in vitro study. J. Ethnopharmacol..

[34] McKillup S. (2012). Statistics Explained: An Introductory Guide for Life Scientists.

[35] Acharya D., Enslin G., Chen W., Sandasi M., Mavimbela T., Viljoen A. (2014). A chemometric approach to the quality control of *Sutherlandia* (cancer bush). Biochem. Syst. Ecol..

[36] Gertsch J. (2009). How scientific is the science in ethnopharmacology? Historical perspectives and epistemological problems. J. Ethnopharmacol..

[37] Street R.A., Prinsloo G. (2013). Commercially Important Medicinal Plants of South Africa: A Review. J. Chem..

[38] Chinkwo K.A. (2005). *Sutherlandia frutescens* extracts can induce apoptosis in cultured carcinoma cells. J. Ethnopharmacol..

[39] Stander A., Marais S., Stivaktas V., Vorster C., Albrecht C., Lottering M., Joubert A.M. (2009). In vitro effects of *Sutherlandia frutescens* water extracts on cell numbers, morphology, cell cycle progression and cell death in a tumorigenic and a non-tumorigenic epithelial breast cell line. J. Ethnopharmacol..

[40] Skerman N.B., Joubert A.M., Cronje M.J. (2011). The apoptosis inducing effects of *Sutherlandia* spp. extracts on an oesophageal cancer cell line. J. Ethnopharmacol..

[41] Leishing G., Loos B., Nell T., Engelbrecht A.M. (2015). *Sutherlandia frutescens* treatment induces apoptosis and modulates the PI3-kinase pathway in colon cancer cells. S. Afr. J. Bot..

[42] Hasjim J., Lavau G.C., Gidley M.J., Gilbert R.G. (2010). In Vivo and In Vitro Starch Digestion: Are Current in Vitro Techniques Adequate?. Biomacromolecules.

[43] Butterworth P.J., Warren F.J., Ellis P.R. (2011). Human a-amylase and starch digestion: An interesting marriage. Starch.

[44] Tundis R., Loizzo M.R., Menichini F. (2010). Natural products as alpha-amylase and alpha-glucosidase inhibitors and their hypoglycaemic potential in the treatment of diabetes: An update. Mini Rev. Med. Chem..

[45] Unoki H., Bujo H., Yamagishi S., Takeuchi M., Imaizumi T., Saito Y. (2007). Advanced glycation end products attenuate cellular insulin sensitivity by increasing the generation of intracellular reactive oxygen species in adipocytes. Diabetes Res. Clin. Pract..

[46] Wu C.H., Huang H.W., Huang S.M., Lin J.A., Yeh C.T., Yen G.C. (2011). AGE-induced interference of glucose uptake and transport as a possible cause of insulin resistance in adipocytes. J. Agric. Food Chem..

[47] Pinto-Junior D.C., Silva K.S., Michalani M.L., Yonamine C.Y., Esteves J.V., Fabre N.T., Thieme K., Catanozi S., Okamoto M.M., Seraphim P.M. (2018). Advanced glycation end products induced insulin resistance involves repression of skeletal muscle GLUT4 expression. Sci. Rep..

[48] Rogero M.M., Calder P.C. (2018). Obesity, Inflammation, Toll-Like Receptor 4 and Fatty Acids. Nutrients.

[49] Mazibuko-Mbeje S.E., Dludla P.V., Roux C., Johnson R., Ghoor S., Joubert E., Louw J., Opoku A.R., Muller C.J.F. (2019). Aspalathin-enriched green rooibos extract reduces hepatic insulin resistance by modulating PI3K/AKT and AMPK pathways. Int. J. Mol. Sci..

[50] Gao Q., Wang X., Zhou J., Fan J. (2011). Cell line misidentification: The case of the Chang liver cell line. Hepatology.

[51] Dang N.T., Mukai R., Yoshida K.-I., Ashida H. (2010). D-pinitol and myo-inositol stimulate translocation of glucose transporter 4 in skeletal muscle of C57BL/6 mice. Biosci. Biotechnol. Biochem..

[52] Lui T.-N., Tsao C.-W., Huang S.-Y., Chang C.-H., Cheng J.-T. (2010). Activation of imidazoline I2B receptors is linked with the AMP-kinase pathway to increase glucose uptake in cultured C2C12 cells. Neurosci. Lett..

[53] Miller A.R., Etgen G.J. (2003). Novel peroxisome proliferator-activated receptor ligands for Type 2 diabetes and the metabolic syndrome. Expert Opin. Investig. Drugs.

[54] Burgermeister E., Schnoebelen A., Flament A., Benz J., Stihle M., Gsell B., Rufer A., Ruf A., Kuhn B., Märki H.P. (2006). A novel partial agonist of peroxisome proliferator-activated receptor-gamma (PPARgamma) recruits PPARgamma-coactivator-1alpha, prevents triglyceride accumulation, and potentiates insulin signaling in vitro. Mol. Endocrinol..

[55] Sorisky A. (2017). Effect of high glucose levels on white adipose cells and adipokines—Fuel for the fire. Int. J. Mol. Sci..

[56] Cernea S., Dobreanu M. (2013). Diabetes and beta cell function: From mechanisms to evaluation and clinical implications. Biochem. Med..

[57] Mncwangi N.P., Viljoen A.M. (2012). Quantitative variation of amino acids in *Sutherlandia frutescens* (Cancer bush)—Towards setting parameters for quality control. S. Afr. J. Bot..

[58] Purwana I., Zheng J., Li X., Deurloo M., Son D.O., Zhang Z., Liang C., Shen E., Tadkase A., Feng Z.P. (2014). GABA promotes human β-cell proliferation and modulates glucose homeostasis. Diabetes.

[59] Gerber P.A., Rutter G.A. (2017). The role of oxidative stress and hypoxia in pancreatic beta-cell dysfunction in diabetes mellitus. Antioxid. Redox Signal..

[60] Jiang J., Chuang D.Y., Zong Y., Patel J., Brownstein K., Lei W., Lu C.-H., Simonyi A., Gu Z., Cui J. (2014). *Sutherlandia frutescens* Ethanol Extracts Inhibit Oxidative Stress and Inflammatory Responses in Neurons and Microglial Cells. PLoS ONE.

[61] Tobwala S., Fan W., Hines C.J., Folk W.R., Ercal N. (2014). Antioxidant potential of *Sutherlandia frutescens* and its protective effects against oxidative stress in various cell cultures. BMC Complement. Altern. Med..

[62] Sia C. (2004). Spotlight on Ethnomedicine: Usability of *Sutherlandia frutescens* in the Treatment of Diabetes. Rev. Diabet. Stud..

[63] Szkudelski T. (2001). The mechanism of alloxan and streptozotocin action in B cells of the pancreas. Physiol. Res..

[64] Chang C.-H., Cheng J.-T. (2015). Canavanine activates imidazoline I-2 receptors to reduce hyperglycaemia in type 1-like diabetic rats. Chem. Biol. Interact..

[65] Lin M.-H., Hsu C.-C., Lin J., Cheng J.-T., Wu M.C. (2017). Identification of morin as an agonist of imidazoline I-3 receptor for insulin secretion in diabetic rats. Naunyn-Schmiedeberg’s Arch. Pharmacol..

[66] Al-Nimer M.S.M. (2017). Drugs acting on imidazoline receptor are still in infancy for treating the Diabetes Mellitus. Int. J. Pharma. Drug Dev.

[67] Nazaruk J., Borzym-Kluczyk M. (2014). The role of triterpenes in the management of diabetes mellitus and its complications. Phytochem. Rev..

[68] Hamid K., Alqahtani A., Kim M.-S., Cho J.-L., Cui P.H., Li C.G., Groundwater P.W., Li G.Q. (2015). Tetracyclic triterpenoids in herbal medicines and their activities in diabetes and its complications. Curr. Top. Med. Chem..

[69] Kunt T. (2001). Current strategies for controlling postprandial hyperglycaemia. Int. J. Clin. Pract. Suppl..

[70] Carroll M.F., Izard A., Rinoni K., Burge M.R., Schade D.S. (2002). Control of Postprandial Hyperglycemia: Optimal use of short-acting insulin secretagogues. Diabetes Care.

[71] Lei W., Browning J.D., Eichen P.A., Folk W.R., Sun G.Y., Lubahn D.B., Fritsche K.L. (2016). An investigation into the immunomodulatory activities of *Sutherlandia frutescens* in healthy mice. PLoS ONE.

[72] Ajit D., Simonyi A., Li R., Chen Z., Hannink M., Fritsche K.L., Mossine V.V., Smith R.E., Dobbs T.K., Luo R. (2016). Phytochemicals and botanical extracts regulate NF-κB and Nrf2/ARE reporter activities in DI TNC1 astrocytes. Neurochem. Int..

[73] Sun Y., Mehmood A., Giampieri F., Battino M.A., Chen X. (2024). Insights into the cellular, molecular, and epigenetic targets of gamma-aminobutyric acid against diabetes: A comprehensive review on its mechanisms. Crit. Rev. Food Sci. Nutr..

[74] Hopkins A.L. (2008). Network pharmacology: The next paradigm in drug discovery. Nat. Chem. Biol..

[75] Noor F., Tahir ul Qamar M., Ashfaq U.A., Albutti A., Alwashmi A.S.S., Aljasir M.A. (2022). Network Pharmacology Approach for Medicinal Plants: Review and Assessment. Pharmaceuticals.

[76] Kifle Z.D., Yesuf J.S., Atnafie S.A. (2020). Evaluation of in vitro and in vivo Anti-Diabetic, Anti-Hyperlipidemic and Anti-Oxidant Activity of Flower Crude Extract and Solvent Fractions of *Hagenia abyssinica* (Rosaceae). J. Exp. Pharmacol..

[77] Van L.V., Pham E.C., Nguyen C.V., Nguyen Duong N.T., Thi T.V.L., Truong T.N. (2023). In vitro and in vivo antidiabetic activity, isolation of flavonoids, and in silico molecular docking of stem extract of *Merremia tridentata* (L.). Biomed. Pharmcacother..

