# Supplementary material for: Comprehensive Mechanistic Characterisation of the Antidiabetic Profile of Sutherlandia frutescens Using Target-Directed In Vitro Assays and Cellomics
Source: Life (Basel). 2026 Aug 17;16(8):1348. doi: 10.3390/life16081348 (PMC13514425; doi:10.3390/life16081348)
Supplement: Supplementary file 1 [file life-16-01348-s001.zip › Figure S1.pdf]

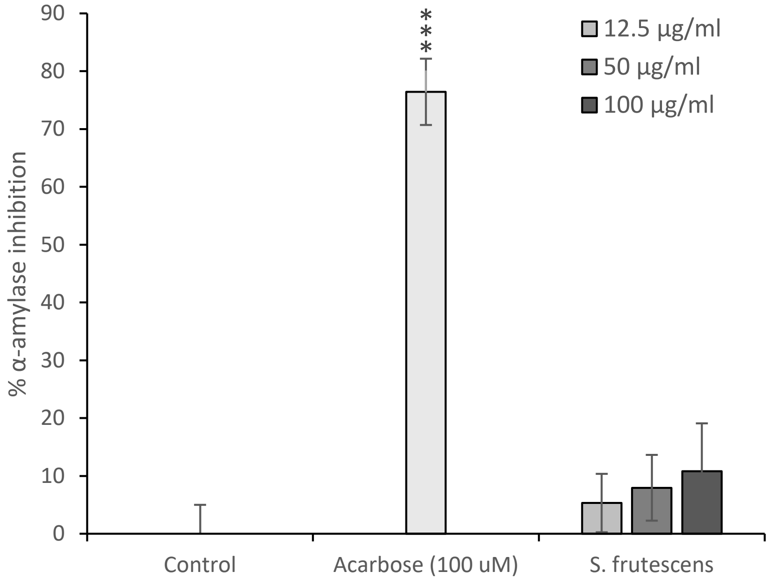

Figure S1: The α-amylase inhibitory activities of *S. frutescens*. Acarbose was included as a positive control. Results are reported as the mean ± standard deviation (error bars shown in black) with all experiments performed three times, each in triplicate (ANOVA and Tukey HSD post-hoc test: *$p<0.05$; **$p<0.01$; ***$p<0,005$ compared to control).
