# Supplementary material for: Comprehensive Mechanistic Characterisation of the Antidiabetic Profile of Sutherlandia frutescens Using Target-Directed In Vitro Assays and Cellomics
Source: Life (Basel). 2026 Aug 17;16(8):1348. doi: 10.3390/life16081348 (PMC13514425; doi:10.3390/life16081348)
Supplement: Supplementary file 1 [file life-16-01348-s001.zip › Figure S2.pdf]

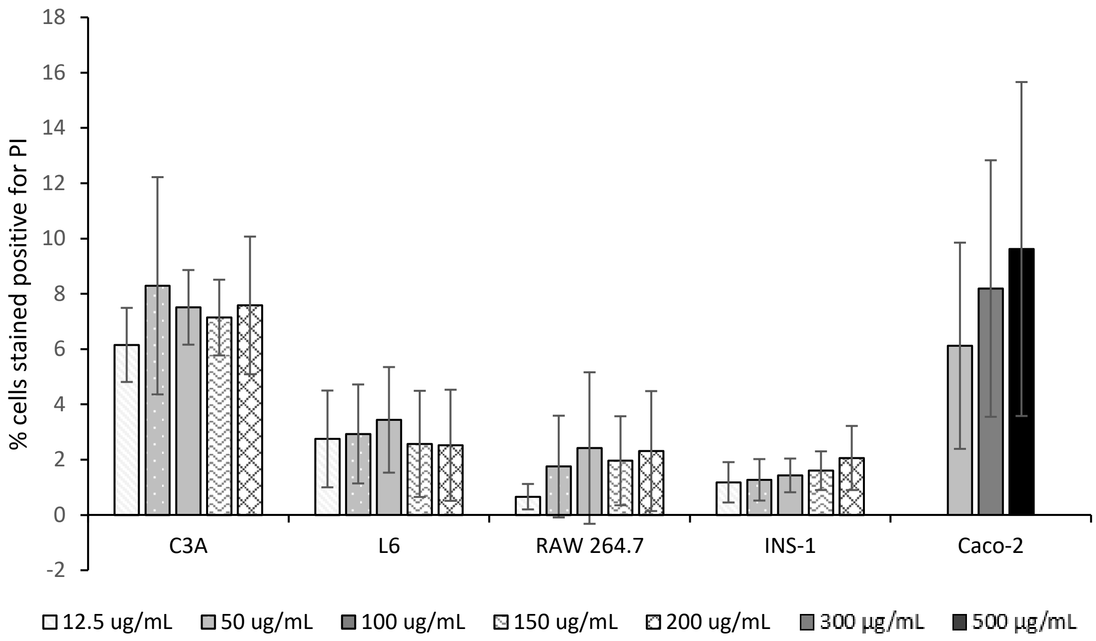

Figure S2: Basal cytotoxicity screening of *S. frutescens* hot aqueous extract comparing cells stained positive for propidium iodide (PI) staining for C3A hepatocytes after 48 hours, differentiated L6 myotubes after 24 hours, RAW 264.7 macrophages in the presence of LPS after 48 hours, INS-1 pancreatic β-cells after 24 hours and Caco-2 cells after 3 hours. Results are reported as the mean ± standard deviation (error bars shown in black) with all experiments performed three times, each in triplicate (ANOVA and Tukey HSD post-hoc test: *p<0.05, **p<0.01, ***p<0.005 compared to control).
