# Supplementary material for: Comprehensive Mechanistic Characterisation of the Antidiabetic Profile of Sutherlandia frutescens Using Target-Directed In Vitro Assays and Cellomics
Source: Life (Basel). 2026 Aug 17;16(8):1348. doi: 10.3390/life16081348 (PMC13514425; doi:10.3390/life16081348)
Supplement: Supplementary file 1 [file life-16-01348-s001.zip › Figure S3.pdf]

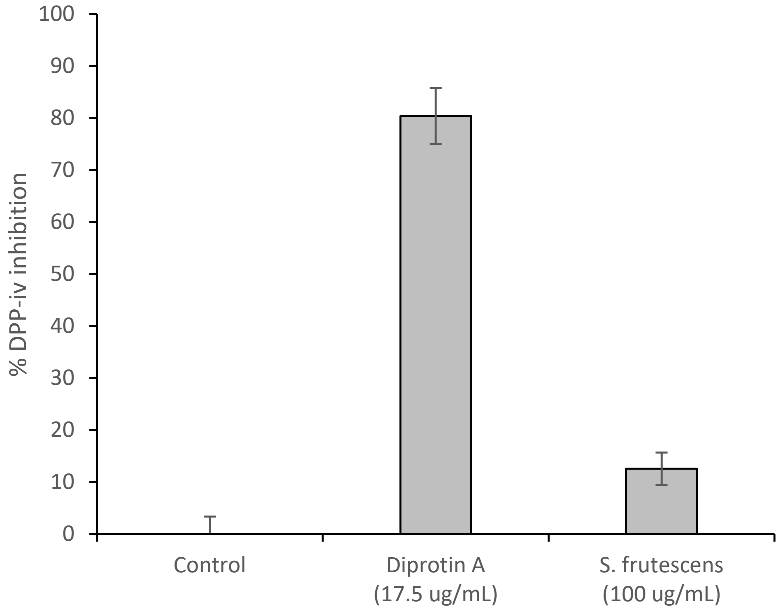

Figure S3: The DPP-iv inhibitory activity of *S. frutescens*. Diprotin A was included as a positive control. Results are reported as the mean ± standard deviation (error bars shown in black) with the experiment performed once in triplicate.
