# Supplementary material for: Comprehensive Mechanistic Characterisation of the Antidiabetic Profile of Sutherlandia frutescens Using Target-Directed In Vitro Assays and Cellomics
Source: Life (Basel). 2026 Aug 17;16(8):1348. doi: 10.3390/life16081348 (PMC13514425; doi:10.3390/life16081348)
Supplement: Supplementary file 1 [file life-16-01348-s001.zip › Table S1.pdf]

TABLE S1: Summary of the *in vitro* screening assays and the respective outcomes (Pringle *et al.*).

Assay	Model	Treatment duration	Treatment concentration	Positive control	Primary endpoint	Main result for *S. frutescens*
α-Amylase inhibition	Porcine pancreatin starch hydrolysis	Direct inhibition	0-500µg/ml	Acarbose	Starch hydrolysis	No inhibition. Data not shown in text.
α-Glucosidase inhibition (sucrase)	Rat intestinal acetone extract. Sucrose as substrate	Direct inhibition	0-500µg/ml	Acarbose	Glucose release from sucrose	Significant ($p<0.01$ and $p<0.005$) at all tested concentrations. Dose dependent trend with maximum inhibition <30%. Weak inhibition relative to the positive control Acarbose (>80%). Mirrors maltase inhibition.
α-Glucosidase inhibition (maltase)	Rat intestinal acetone extract. Maltose as substrate	Direct inhibition	0-500µg/ml	Acarbose	Glucose release from maltose	Significant ($p<0.05$ and 0.005) at all tested concentrations. Dose dependent response with maximum inhibition < 30%. Weak inhibition relative to the positive control Acarbose (> 80%). Mirrors sucrase inhibition.
Intestinal glucose uptake	Confluent Caco-2 culture	3 Hours	0-500µg/ml	Myricetin	Glucose uptake	Dose dependent inhibition at all tested concentrations. Dose dependent trend with maximum inhibition < 20% ($p<0.05$). Magnitude of response comparable to positive control Myricetin (< 30%).
DPP-iv inhibition	Recombinant Human DPP-iv	Direct inhibition	0-100µg/ml	Diprotin	PNA-release	No meaningful inhibition evident. Data not shown in text.
Protein glycation inhibition	AGE formation using BSA as surrogate protein	30 Hours 60°C	0-200µg/ml	Aminoguanidine	Fluorescent AGE	Dose dependent inhibition ($p<0.05$ and $p<0.005$), weak relative to positive control.
Hepatic glucose utilisation	C3A hepatocytes	48 Hours	0-100µg/ml	Metformin	Glucose	Dose dependent increase in glucose consumption at 50-100µg/ml ($p<0.01$ and $p<0.005$). Effect comparable to response from positive control metformin.

Hepatic lipid accumulation	Palmitic acid induced lipid accumulation in C3A hepatocytes	24 Hours	0-100µg/ml	Metformin	Neutral lipid content	No meaningful response. No significant inhibition.
Muscle glucose utilisation	C2C12 myotubes	3 Hours	0-100µg/ml	Metformin	Glucose	Dose dependent increase in glucose consumption, but effect appears saturated after 50µg/ml ($p<0.005$). Response magnitude comparable to positive control metformin.
Muscle lipid accumulation	Palmitic acid induced lipid accumulation in C2C12 myotubes	24 Hours	0-100µg/ml	Metformin	Neutral lipid content	Small, statistically insignificant ($p>0.05$) inhibition of lipid. No clear dose dependent trend evident and response weak relative to magnitude of positive control metformin.
Pancreatic lipase inhibition	Porcine pancreatin lipase activity	Direct inhibition	0-500µg/ml	Orlistat	PNA release from pNP-Palmitate	No meaningful inhibition.
Macrophage activation/function	COX-2 levels in LPS activated RAW 264.7 macrophages	24 Hours	0-200µg/ml	Resveratrol	Antibody staining (COX-2)	Dose dependent decrease, but only 100µg/ml statistically significant ($p<0.05$).
Macrophage activation/function	iNOS levels in LPS activated RAW 264.7 macrophages	24 Hours	0-100µg/ml	Resveratrol	Antibody staining (iNOS)	Dose dependent decreasing trend, but not statistically significant ($p>0.05$).
Macrophage activation/function	NFκB activation in LPS activated RAW 264.7 macrophages	24 Hours	0-100µg/ml	LPS	Antibody staining (phosphor-NF-κB)	Dose dependent decrease, but only 100µg/ml statistically significant ($p<0.005$).
Insulin release (calcium signalling)	Calcium levels in INS-1 cells	24 Hours	0-100µg/ml	Tolbutamide	Cellular calcium flux. Fluorescent probe Fluo-4	Dose dependent trend, but only 100µg/ml statistically significant ($p<0.05$). Magnitude of response weak relative to positive control tolbutamide.

Antioxidant	FRAP	Direct reaction	0-100µg/ml	Catechin	Trolox equivalents	Weak direct antioxidant activity relative to positive control.
Antioxidant	DPPH radical scavenging	Direct reaction	0-100µg/ml	Catechin	DPPH radical scavenging	Weak direct antioxidant activity relative to positive control.
Pancreatic β-Cell proliferation	INS-1 proliferation	24-Hours	0-100µg/ml	GABA	Cell density based on nuclear counts (Hoechst)	Dose dependent trend but no statistical significance reached. Response magnitude comparable to positive control GABA.
Pancreatic β-Cell antioxidant capacity	TBHP induced oxidative stress in INS-1 cells	24 Hours	0-50µg/ml	Catechin	ROS levels based of fluorescent probe CellROX orange	No meaningful enhancement of cellular antioxidant activity evident.
Pancreatic β-Cell resistance to oxidative stress	INS-1 survival against TBHP induced oxidative stress	24 Hours	0-50µg/ml	Catechin	Cell density based on nuclear counts (Hoechst)	Increased cell survival. Significant at 12.5 and 50µg/ml ($p<0.01$ and $p<0.05$). Response magnitude comparable to positive control.
Adipogenesis	Glycerol-3-phosphate dehydrogenase activity in 3T3-L1 cells	72-Hours	0-250µg/ml	Rosiglitazone	G3PDH activity (adipogenesis marker)	No increase in G3PDH activity indicating no direct adipogenic potential.
Adipocyte lipogenesis	Lipid accumulation in PPAR activated 3T3-L1 adipocytes	72 Hours	0-250µg/ml	GW 9662	Triglyceride content	Dose dependent decline in PPAR-γ stimulated triglyceride accumulation. Antagonistic toward PPAR-γ activation.
